# Incorporating Prior Knowledge in the Seeds of Adaptive Sampling Molecular Dynamics Simulations of Ligand Transport in Enzymes with Buried Active Sites

**DOI:** 10.1021/acs.jctc.4c00452

**Published:** 2024-07-09

**Authors:** Dheeraj Kumar Sarkar, Bartlomiej Surpeta, Jan Brezovsky

**Affiliations:** † Laboratory of Biomolecular Interactions and Transport, Department of Gene Expression, Institute of Molecular Biology and Biotechnology, Faculty of Biology, Adam Mickiewicz University, Uniwersytetu Poznanskiego 6, Poznan 61-614, Poland; ‡ International Institute of Molecular and Cell Biology in Warsaw, Ks Trojdena 4, Warsaw 02-109, Poland

## Abstract

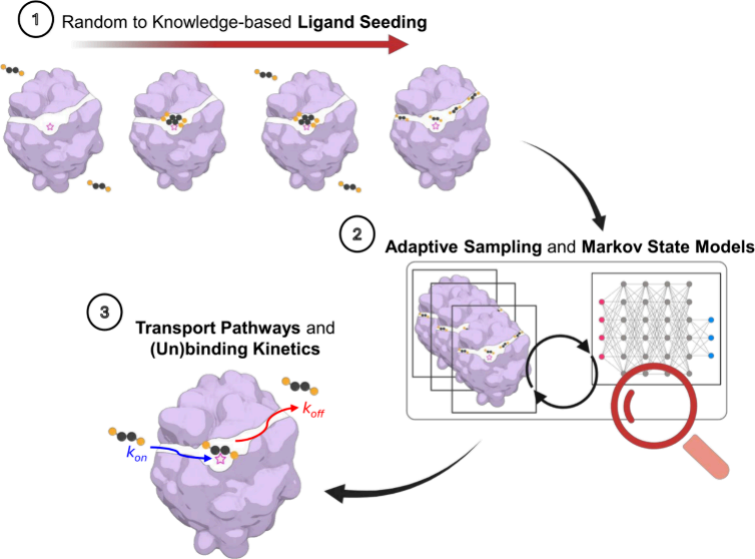

Because most proteins have buried active sites, protein tunnels or channels play a crucial role in the transport of small molecules into buried cavities for enzymatic catalysis. Tunnels can critically modulate the biological process of protein–ligand recognition. Various molecular dynamics methods have been developed for exploring and exploiting the protein–ligand conformational space to extract high-resolution details of the binding processes, a recent example being energetically unbiased high-throughput adaptive sampling simulations. The current study systematically contrasted the role of integrating prior knowledge while generating useful initial protein–ligand configurations, called seeds, for these simulations. Using a nontrivial system of a haloalkane dehalogenase mutant with multiple transport tunnels leading to a deeply buried active site, simulations were employed to derive kinetic models describing the process of association and dissociation of the substrate molecule. The most knowledge-based seed generation enabled high-throughput simulations that could more consistently capture the entire transport process, explore the complex network of transport tunnels, and predict equilibrium dissociation constants, *k*_*off*_*/k*_*on*_, on the same order of magnitude as experimental measurements. Overall, the infusion of more knowledge into the initial seeds of adaptive sampling simulations could render analyses of transport mechanisms in enzymes more consistent even for very complex biomolecular systems, thereby promoting drug development efforts and the rational design of enzymes with buried active sites.

## 1 Introduction

Molecular recognition is critical for all biological processes. Therefore, it has been a long-standing quest to capture the intrinsically dynamic and volatile nature of protein–ligand (un)binding processes by high-resolution sampling and resolve the meaningful kinetics of ligand binding processes in structure-based drug design.^1,2^ Additionally, a ligand can prefer multiple routes of entry to interact with the environment of an active site.^3−5^ These routes, often referred to as tunnels, are seen to have equivalent importance as the catalytic properties of enzymes.^6^ Whereas in the majority of enzymes, the active site is buried,^7,8^ the underlying molecular properties of the tunnels can control the entry and exit of ligands to a greater extent, specifically via gating residues.^9^ In this context, the ligand binding processes via those transport pathways are a critical component in biocatalysis and for identifying critical residues underlying the transport processes for mutagenesis and rational drug design.^6,10^ Hence, protein tunnels are well-placed when considering improved catalysis and features such as the specificity and altered activity of small molecules. Very often, tunnel lining residues or other gating residues can act as hot spots other than active site residues.^9,10^

Transport processes, such as the migration of ligands from an active site to the bulk solvent, are often connected with the requirement of overcoming a high energy barrier, resulting in the rare nature of such an event.^11^ Because molecular dynamics (MD) simulations can observe biologically relevant processes even at atomistic resolution, they are extensively used to study the mechanisms, dynamics, and functions of biomolecular complexes.^12,13^ Numerous computational approaches have been developed in recent years to sample such rare events of ligand transport processes involving the association and dissociation of ligands and receptors.^14^ These approaches benefit from the improvement of computational hardware in terms of GPUs as well as the implementation of various path sampling methods and methods for sampling rare events.^15,16^ Specifically, enhanced sampling methods such as milestoning,^17^ weighted ensemble,^18^ Gaussian accelerated MD,^19,20^ metadynamics,^21,22^ adaptive sampling MD (ASMD) based on Markov state models (MSM),^5,23,24^ and random acceleration MDs^5,25,26^ have gained popularity in studying such rare events. Although most methods use additional potential or force to bias the simulations along a designed collective variable, ASMD methods utilizing MSMs can avoid such perturbations.^27−29^ MSMs is an approach for extracting kinetic information from unbiased MD simulations, capable of deriving longer-time scale insights from shorter trajectories. MSM modeling entails the collection of data from the trajectory set, followed by the decomposition of the molecular ensemble into microstates, which are subsequently employed to enumerate transitions between them. This enables the construction of a transition probability matrix and definition of metastable states, which can then be used to create a final model, delivering insights into the conditional probabilities of being in a particular state and transitioning to another state at a specific time (also known as lag time). In simple terms, the MSM can be described as a network, in which the metastable states are represented as nodes, while the edges represent the transition rates between those states.^29−31^

Extensive ASMD simulations have been successfully used to study ligand binding processes.^5,24,32−35^ ASMD is an energetically unbiased protocol comprising iterative rounds of intelligently respawned equilibrium simulations of protein–ligand configurations. The procedure starts with generating the set of initial configurations, which are called seeds. From these seeds, a set of independent simulation runs is performed, and preliminary MSM is created from these simulations. Next, a scoring function is used to identify the least explored, most informative metastates in this MSM, employed to respawn the subsequent batch of simulations.^28,29^ The whole ASMD procedure consists of several iterative rounds (called epochs) of MD simulations, data aggregation from all previous simulations, an MSM creation, metastate scoring, and the generation of new starting configurations. Usually, ASMD setup speeds sampling by about an order of magnitude over the standard MD simulations.^33,36,37^ However, ASMDs are still ineffective when a too large barrier is encountered that cannot be overcome in an unbiased regimen, requiring the sampling of the ensuing rare events with enhanced sampling methods along predefined collective variable, and incorporating such data through the concept of multiensemble MSM.^38^ Finally, most ASMD protocols suffer from exploitation-exploration trade-off,^39^ meaning that already visited states will less likely be respawn for simulations in the next epochs, potentially limiting the sampling of some metastable states required for accurate description of processes.^35^ Given the rising success of ASMD simulations in ligand transport studies, the impact of designing individual components in an ASMD workflow on the efficacy of sampling relevant regions of the protein–ligand configurational space is of interest.^24,33,35,40,41^ Betz and Dror investigated the role of a scoring function for selecting the configuration for successive iterations to partially overcome the exploration–exploitation trade-off using the well-known test system of trypsin with a benzamidine inhibitor and a more complex yet realistic system of membrane-bound adrenergic receptor β_2_ with dihydroalprenolol inhibitor.^35^ They compared three scoring functions: simple counts, in which states were resampled with a probability inversely proportional to their occurrence in the simulation; population scores, which prefer states with smaller populations in MSMs; and hub scores, which select states with lower connectivity in MSMs, as the measure of connectivity of states in MSMs. For that membrane-bound system, the count score could not govern the ASMD toward investigating inhibitor migration through the protein, by focusing entirely on the membrane region. In contrast, the other two scores successfully sampled the relevant configurations. Hence, the use of more information-rich functions markedly benefited the study of ligand transport in that more complex setting.

**Figure 1:**
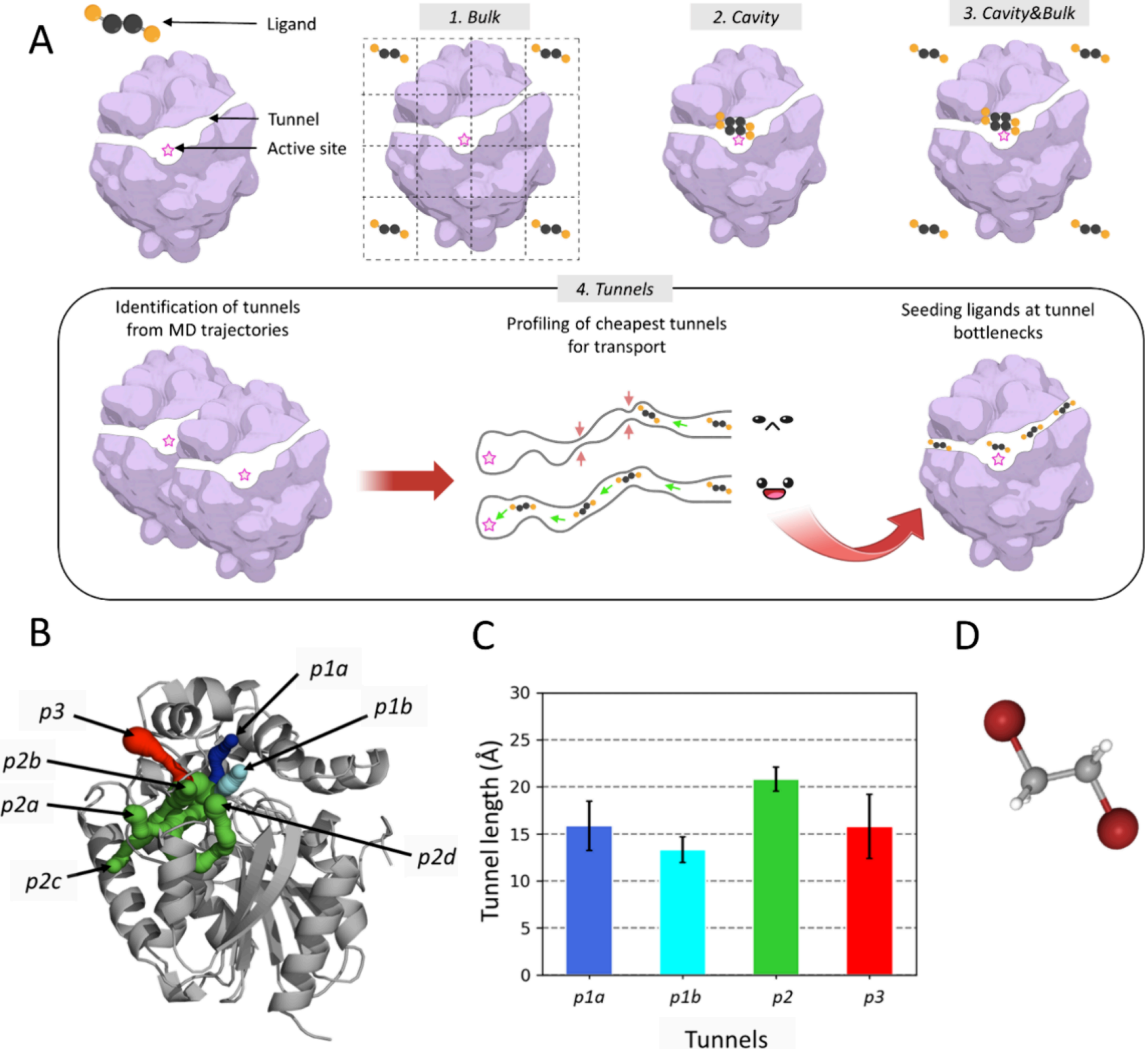
Overview of the evaluated seeding schemes and model system used. (A) Schematic representation of the studied schemes and seeding of the substrate molecule from random to knowledge-based positions. (B) Representative structure of tunnel network from 100 ns MD simulation of LinB86 (see Table S1 for other tunnel properties). The known tunnels are shown as sets of colored spheres: *p1a* (blue), *p1b* (cyan), four branches of *p2* (green), and *p3* (red). The protein structure is shown as a gray cartoon. (C) Average lengths of ensembles of the known tunnels observed in the MD simulation. The data represent mean ± stdev from the simulation. (D) A structure of 1,2-dibromoethane (DBE), which is the substrate of haloalkane dehalogenase LinB86.

Here, we investigated the role of employing relevant information while preparing the initial seeding structures for ASMD. We designed four schemes (Figure 1A) from a random positioning of the ligand around the protein to more knowledge-based poses of the ligand bound in the active site or along the tunnels precomputed from the apo simulation. We tested the capabilities of ASMD, initiated from these seeding schemes, in exploring and exploiting the transport tunnels in haloalkane dehalogenase mutant LinB86 (Figure 1B,C), in which an additional functional tunnel was introduced *de novo*.^10^ By performing intensive ASMD simulations of LinB86 with one of its substrates, 1,2-dibromoethane (DBE) (Figure 1D), for each scheme, we were able to compare to what degree the initial seeding impacted the ability of ASMD to (i) capture the entire process of substrate association and dissociation, (ii) consistently identify the metastable states adopted by substrate, (iii) predict kinetic parameters of the process, and finally (iv) describe the complexity of transport via multiple transport tunnels.

## 2 Materials and Methods

### 2.1 Seed Generation for ASMD Simulations

The input model was based on the crystallographic structure of the mutant of haloalkane dehalogenase enzyme LinB86 (PDB code: 5LKA).^10^ The protein structure was further protonated using H++ web server^42,43^ at pH 8.5. The protein was modeled using the AMBER ff14SB^42^ force field and the substrate DBE with the General Amber Force Field – GAFF.^44,45^ The partial atomic charges on the DBE molecule were derived using multiconformational, multiorientational restrained electrostatic potential fit.^46^ Each DBE conformation was geometry optimized at the MP2/6-31G(d) level of theory, and its multiorientational molecular electrostatic potential was calculated at the HF/6-31G(d) level using Gaussian v09.^47^ Finally, two-stage charge fitting was conducted for all conformations and orientations using the *resp* and *antechamber* modules of AMBERTools18.^48^

The substrate molecule was placed according to the four designed schemes to investigate systematically the role of prior knowledge in ASMD seeding (Figure 1A). DBE was placed at 30 different positions, to seed 30 independent simulations for each scheme (Figure S1) as follows. (i) In the *Bulk* scheme, DBE was positioned on an equally spaced grid in the solvent surrounding the protein using the “*drawgridbox [selection], nx = 5, ny = 5, nz = 5, padding = 5, lw = 1, r = 0, g = 0*” function of PyMOL.^49^ (ii) In the *Cavity* scheme, DBE was docked to the enzyme’s active site using AutoDock Vina.^50^ The 30 docked poses were derived by defining the grid box centered at the center of mass (COM) of catalytic residues (N38, D108, W109, and H272) with a dimension of 22.5 Å and exhaustiveness of 1000. (iii) In *the Cavity&Bulk* scheme, 15 DBE positions were taken from the *Cavity* scheme based on their energies and first 15 DBE positions were taken from the *Bulk* scheme. Finally, (iv) in the *Tunnels* scheme, putative transport tunnels in LinB86 were detected from the 100 ns trajectory of ligand-free LinB86 simulation, and then the most open tunnels were explored for binding of DBE molecules along these tunnels. Finally, the composite tunnels, formed from parts of tunnels with conformations ensuring minimal energy costs for DBE migration, were generated (see Text S1 and Figures S2–S9 for details of this protocol).

The generated protein–ligand complexes were then solvated based on the 3D reference interaction site model theory^51^ using the Placevent^52^ algorithm. Such a system was then processed with the *tleap* module of AMBERTools18, placing the presolvated proteins in the octahedral box of OPC water molecules^53^ at a distance of 10 Å and neutralizing them with counterions (Na^+^ and Cl^–^) at an ionic strength of 0.1 M. Finally, the hydrogen mass repartitioning (HMR) method was applied to produce topologies to enable a 4 fs time step.^54^ Here, we would like to note that the application of HMR might alter the time scales of the studied processes, as shown recently.^55,56^ Hence, in studies aimed at measuring the absolute rates, unlike the primarily comparative work here, it might be advisable to avoid HMR.

### 2.2 Equilibration MD Simulations of Seeds

The systems were then energy-minimized and equilibrated using PMEMD and PMEMD.CUDA modules^57^ of AMBER18,^48^ respectively. All complexes were energy minimized in five consecutive stages, each composed of 100 steps of the steepest descent followed by 400 steps of the conjugate gradient method, with gradually decreasing restraints on the protein atoms (initially 500 to heavy atoms, and later restraints of 500, 125, 25, and 0.001 kcal.mol^–1^·Å^–2^ applied only to the backbone atoms). Minimization was followed by 20 ps heating from 0 to 200 K in the NVT ensemble using the Langevin thermostat^59^ with a collision frequency of 2 ps^–1^ and coupling constant of 1 ps while keeping the protein heavy atoms restrained with a force constant of 5 kcal·mol^–1^·Å^–2^. Next, the temperature was raised to the target value of 310 K during the first 100 ps of the NVT simulation and kept constant for 900 ps, employing the same parameters as previously described. This was followed by an NPT simulation at 1 atm enforced by the weak-coupling barostat with a coupling constant of 1 ps using positional restraints of 5 kcal·mol^–1^·Å^–2^ on the backbone atoms for 1 ns, followed by 1 ns without any positional restraints. All MD simulation stages were run using a 4 fs time step enabled by SHAKE^58^ and HMR algorithms, periodic boundary conditions, and particle mesh Ewald method,^60^ with the nonbonded cutoff of 8 Å. The trajectories were generated by saving coordinates every 20 ps. The MD trajectories were analyzed using the *cpptraj* module of AMBERTools23,^61,62^ The last snapshots from the unrestrained simulation were used as the initial input structures for ASMD.

### 2.3 High Throughput ASMD to Study Substrate Un(binding) Processes

ASMD was set up with 30 epochs, each consisting of 30 separate production simulations. To build an MSM after each epoch, we used the distances between the Cα atoms of the protein and four heavy atoms of DBE and reduced the high dimensional space to three dimensions using time-lagged independent component analysis (TICA)^63^ with a lag time of 2 ns. ASMD simulations were performed using HTMD v1.13.10^27^ and AMBER18^48^ software packages. The equilibration phase in HTMD consisted of two 250 ps NVT and NPT simulations, during which the systems were heated from 0 to 310 K with a Langevin thermostat and harmonic positional restraints to the backbone atoms with a force constant of 5 kcal·mol^–1^·Å^–2^. Finally, a 50 ns unrestrained production MD was performed in the NVT ensemble using a weak-coupling thermostat and a saving frequency of 100 ps, since only the NVT production simulations were fully supported by HTMD package. Such ASMD runs were performed in three replicates for each investigated seeding scheme.

### 2.4 Final MSM Construction and Validation

All the MSMs were built using HTMD,^27^ which internally uses the PyEMMA program,^64^ following the standard PyEMMA protocols. The high dimensional data from adaptive sampling projected using the distance feature was reduced to three dimensions using TICA^63^ with a lag time of 2 ns. Next, the reduced TICA coordinates were clustered into 1000 microstates using the MiniBatchKMeans^65^ method. The metastable states were lumped using the PCCA++ method,^66^ with the number of metastable states based on spectral analysis^64^ and verified against plots of linear implied time scales (Figures S10–S12). A lag time of 20 ns was used during MSM construction. Finally, the Chapman–Kolmogorov^67^ test was performed to confirm the Markovianity of the generated MSMs (Figures S13–S21).

### 2.5 MSM Analysis and Comparison

In order to quantify the ability of ASMD to sample the whole (un)binding process of DBE, the distances between the COM of the DBE molecule and the COM of three catalytic residues (N38, D108, and W109, Figure S22A) were measured from the ∼900 trajectories for each replicate using the *cpptraj* module of AMBERTools23.^61,62^ Using this distance, we could define the location of DBE in the active site (0–5 Å), tunnel (5–19 Å), and bulk (>19 Å). The cutoff of 19 Å for tunnels was derived from the average lengths of the investigated tunnels measured by CAVER (Figure 1C and Table S1). Finally, the transition path theory approach implemented in PyEMMA was used to derive transition probability matrices and compute the mean first-passage times of each association and dissociation process in MSMs. Here, the metastable states with the most prevalent bound conformation of DBE were used as sink states. In contrast, the metastable states featuring DBE, mainly in the bulk solvent, were considered as source states to perform the transition flux analysis and derive the transition probabilities and kinetics rates. Furthermore, the most frequently occurring bottleneck residues were shortlisted from the CAVER results as follows (Figure S22B–E): *p1a* (D147, F151, and V173), *p1b* (D147, W177, and L248), *p2* (L211 and L248), and *p3* (L143, F151, and I213) and the distance between their COM to the COM of DBE was calculated to assess localization of DBE with respect to these tunnels. The fluctuations in the positions of COM of bottleneck and cavity residues were evaluated to confirm their stability and hence suitability to be used as landmarks (Table S2). While the cavity COM was the most stable, the RMSFs of all other COMs were lower than 1.4 Å, indicating the lack of major distortions in these landmarks during the simulations.

Ensembles of 1000 representative structures of metastable states generated from individual MSMs were clustered to establish the correspondence of these metastable states across the explored schemes. For this purpose, the mean, 25th, 50th, and 75th percentiles were calculated for each set of characteristic distances between DBE and the bottleneck residues as well as the catalytic machinery described above (Figure S22). These were used cumulatively as a vector of 20 variables describing each metastable state. Principal component analysis implemented in the Python scikit-learn library^65^ was used to reduce the dimensionality of each vector. The set of the first three principal components for each metastable state was clustered with HDBSCAN^68^ using a *min_cluster_size* of 2, with the remaining parameters kept at their default values.

### 2.6 Analysis of Substrate Utilization of Tunnels

Time-evolution of distances between DBE and the bottleneck residues as well as the catalytic machinery described above (Figure S22) for the entire set of trajectories was used to estimate the approximate position of the ligand in the context of the tunnel network. By tracking the change of the relative position, the movement through a particular tunnel was assigned, where possible. Therefore, the approximate tunnels’ utilization was estimated across the investigated schemes by analyzing the transition between subsequent positions. The procedure was composed of three stages, as follows.

#### i) Position Assignment

First, the closest bottleneck at a particular frame to the DBE molecule was defined. This information was used to define the approximate length of the closest tunnel (i.e., the distance between the COM of catalytic machinery and the COM of the particular bottleneck). These two distances were contrasted with the distance between the ligand and the catalytic machinery, which altogether resulted in the identification of the approximate ligand position. Importantly, at this point, additional parameters were introduced to classify the ligand position, namely bt_cutoff_along = 2.0 Å defining the region around the bottleneck (i.e., whether the ligand is in the bulk, bottleneck region, or tunnel) and bt_cutoff_across = 5.0 Å, which defines whether the ligand is not too far from the bottleneck horizontally in case it is within the bottleneck region. Given these three distances and introduced cutoffs, the following scenarios and corresponding ligand states were considered

- *Bulk (out_)*: Ligand is further from the active site than the sum of tunnel length and *bt_cutoff_along*.
- *Bottleneck (bt_)*: Ligand is within the bottleneck region, either further than the tunnel length or closer than the tunnel length but within *bt_cutoff_along* and *bt_cutoff_across.*
- *Unknown bottleneck (bt_unknown)*: Ligand is within the bottleneck region, either further than the tunnel length or closer than the tunnel length within *bt_cutoff_along* but exceeding the *bt_cutoff_across.*
- *Inside (in_)*: Ligand distance to catalytic machinery is shorter than the tunnel length reduced by the *bt_cutoff_along.*

#### ii) Transition Detection and Classification

Given the defined states for each frame, the transitions between bulk (*out_*) and interior (*in_*) and vice versa were identified. Transitions via bottleneck regions (*in_bt_out* or *out_bt_in*) were also considered. When a mismatch was detected between assigned tunnel *in_out*/*out_in*, we applied an additional dist_tolerance = 1.0 Å parameter. This parameter defined the tolerance distance that is considered for swapping the classification of one of the sides of the transition, thus promoting the tunnel that was seen in the bottleneck region for scenarios where the intermediate state was seen. The transitions were tracked as follows:

- If the transition occurred from the bulk to inside or from the inside to bulk directly, the transition *in_out*/*out_in* was assigned by applying the *dist_tolerance* for cases where a mismatch between both sides occurred.
- If the ligand moved from the interior to the bottleneck region or from the bulk to the bottleneck region, the transition was not assigned; only the information regarding the temporary state was updated.
- If the temporary state was a bottleneck and the closest tunnel changed, the transition was not assigned; only the bottleneck temporary state was updated.
- If the temporary state was a bottleneck and the ligand moved to the same general state but related to a different tunnel, the transition was not assigned; only the general state was updated.
- If the temporary state was a bottleneck and the ligand moved to the other general state (from *in_* to *out_* or from *out_* to *in_*), the transition was assigned. In addition, information was collected about the bottleneck used by applying the *dist_tolerance* for cases where the mismatch between both sides occurred, thus promoting the tunnel of the assigned transition bottleneck state.

#### iii) Characterization of Tunnel Utilization

Finally, all types of unique transitions were counted across all simulations from each scheme and averaged across three replicates performed for each scheme. Importantly, we applied the following classification to assign transitions to particular categories

- Tunnel (*p1a*, *p1b*, *p2*, and *p3*) – all transitions that passed through the bottleneck of a particular tunnel or the direct transitions *in_out* or *out_in* related to the same tunnel on both sides.
- Mixed–all direct transitions *in_out* or *out_in*, where both sides of transitions differed despite the applied distance tolerance.
- Unknown–all transitions that crossed through the unknown bottleneck.

## 3 Results and Discussion

Overall, ∼900 MD trajectories (450,000 frames) with an aggregated simulation time of 45 μs were produced using ASMD of LinB86-DBE complexes generated for each studied scheme (Table S3). For each scheme, ASMD was performed in three replicates (∼135 μs in total) to evaluate the abilities to consistently describe the entire transport processes, focusing on the convergence among ASMD replicates, the degree of quantitative agreement with experimental data, and the ability to consider transport via all known tunnels. These ASMD simulations featured stable protein conformations, with the most mobile part being unstructured N-terminal region (Figures S23 and S24).

### 3.1 Capturing DBE Association and Dissociation Processes in LinB86

**Figure 2:**
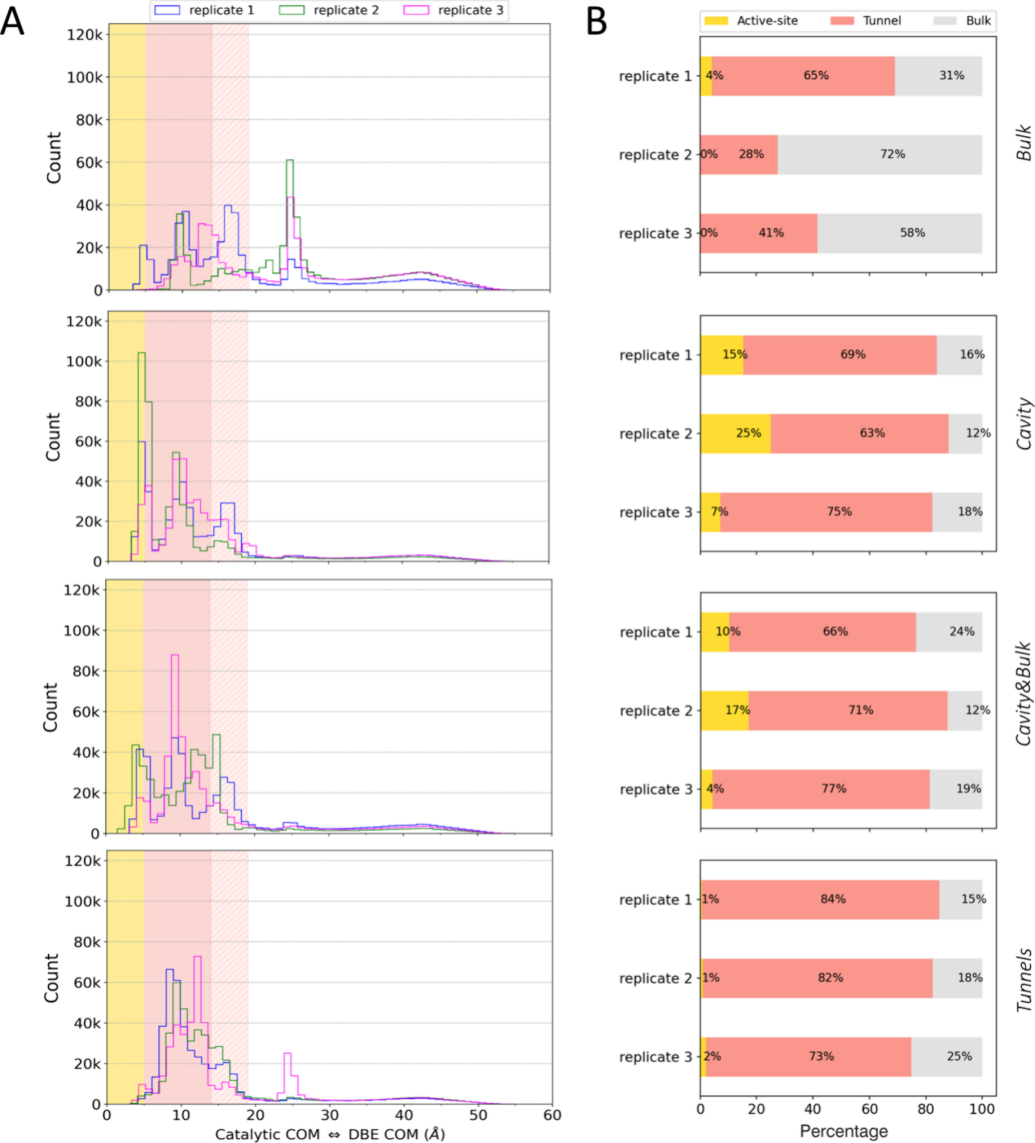
Substrate (un)binding to the active site of LinB86 captured by ASMD simulations with four seeding schemes. (A) The distance distribution of DBE to catalytic residues for three replicated ASMDs for each seeding scheme. The regions corresponding to DBE in the active site (0–5 Å), shortest (*p1b*, 5–14 Å) and longest (*p2*, 14–19 Å) tunnel lengths (Figure 1C), and bulk solvent (>19 Å) are highlighted as gold, pink, shaded pink, and white, respectively. The distances are those between the COM of DBE and the COM of catalytic residues (N38, D108, and W109), measured in 45-μs ASMD simulations. (B) The fraction of DBE seen in individual regions.

**Figure 3:**
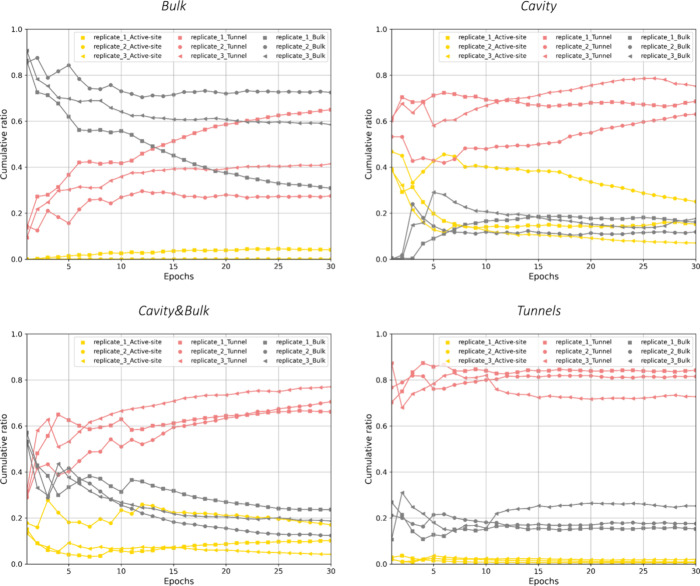
An epoch-wise sampling of the active site, tunnel, and bulk regions of LinB86 by DBE. Each epoch most often comprises 30 separate 50 ns-long production simulations, aggregating about 15,000 frames.

In order to study the applicability of the studied schemes, we initially investigated how effectively each scheme could sample the end points of the processes (i.e., the bound and unbound states of DBE in the active site cavity of LinB86 and bulk solvent, respectively). Those states could be effectively defined by the distance of the DBE COM from the COM of three catalytic residues located at the bottom of the cavity (Figure S22A), defining the bound states within 5 Å distance, whereas the unbound state sampled distances primarily above 19 Å, which were further than the length of the longest tunnels present in LinB86 (Figure 1C). The DBE molecule adopted the unbound state in all schemes and replicates for a substantial fraction of cumulative ASMD trajectories (Figure 2). Curiously, some of the ASMDs, particularly those originating from the *Bulk* scheme but not exclusively, exhibited an area of high probability of DBE occurrence centered at approximately 25 Å-distance from the catalytic residues (Figure 2A). Structurally, this area corresponded to DBE bound at the surface cleft at the C-termini of LinB86 (Figure S25). Even in the *Cavity* scheme initiated from the DBE molecule bound deep in the active site, the substrate reached the bulk solvent, generating a minimum of 12% configurations in the unbound state (Figure 2B). Over 10,000 configurations in the unbound state were generated after at most seven epochs of ASMD simulations (Figure 3). Foreseeably, the unbound states were most prevalent (>31%) in the simulation seeded with DBE placed in the bulk solvent around the enzyme (scheme *Bulk*).

Concerning the ability of ASMDs to reach the bound pose of DBE in the buried active site of LinB86, all schemes except for *Bulk* consistently sampled the bound states in all three replicates. In the case of the *Bulk* scheme, the DBE molecule was able to find a path to the active site in replicate1, producing a total of 4% of simulations in the bound state (Figures 2 and 3), with a significant ensemble of more than 1,000 bound configurations sampled already by the fifth epoch (Figure 3). However, no bound state was observed in the other two replicated ASMDs from the *Bulk* scheme (Figures 2 and 3). This was expected as unbiased simulations of ligand associations are generally rather time-consuming, even for less complex systems.^69−71^

Among the remaining schemes, *Cavity* ASMDs exploited the bound states the most frequently, as expected from the initial seeding with docked poses of DBE (Figure 2). Such a setup led to the accumulation of over 5,000 bound configurations during the first epoch in all three replicates (Figure 3). Such behavior was also partially retained in the *Cavity&Bulk* scheme, where more than 2,000 configurations in the bound state were systematically observed in the first epoch of ASMDs. Here, the additional seeds of DBE placed in the bulk solvent resulted in considerable sampling of more than 10,000 configurations in the unbound state within the first three epochs of ASMDs, about twice as fast as in the pure *Cavity* scheme (Figure 3). Finally, in ASMDs from the *Tunnels* scheme, we observed the most consistent behavior in exploring these three regions, with DBE localized primarily in regions corresponding to transport tunnels (Figures 2 and 3). Given the sufficient coverage of bound and unbound states, we progressed to the creation of MSMs from the assembled trajectories and the calculation of kinetic parameters of descriptions of (un)binding processes. Due to the lack of bound states in the *Bulk* scheme, these ASMDs were not considered for constructing MSMs.

### 3.2 Identifying Metastable States of DBE Interacting with LinB86 and Predicting Kinetic Parameters from MSMs

To further test the capabilities of the studied seeding schemes in the diversity and consistency of the identified metastable states, we generated MSMs from the individual ASMD replicates. These MSMs consisted of three to six metastable states for the *Cavity* (Figures S26–S28) and *Cavity&Bulk* (Figures S29–S31) schemes, whereas six to eight metastable states were identified in MSMs from the *Tunnels* schemes (Figures S32–S34). To understand the mutual correspondence among these states across all generated MSMs, we generated 1,000 representative structures of each metastable state and measured the distances of DBE to the catalytic residues, as well as to the bottlenecks of the known transport tunnels in LinB86 (Figure S22). These distances represent fingerprints characterizing the metastable states (Figures S35–S37), clearly identifying not only unbound and bound states but also their alignment to individual transport tunnels.

**Figure 4:**
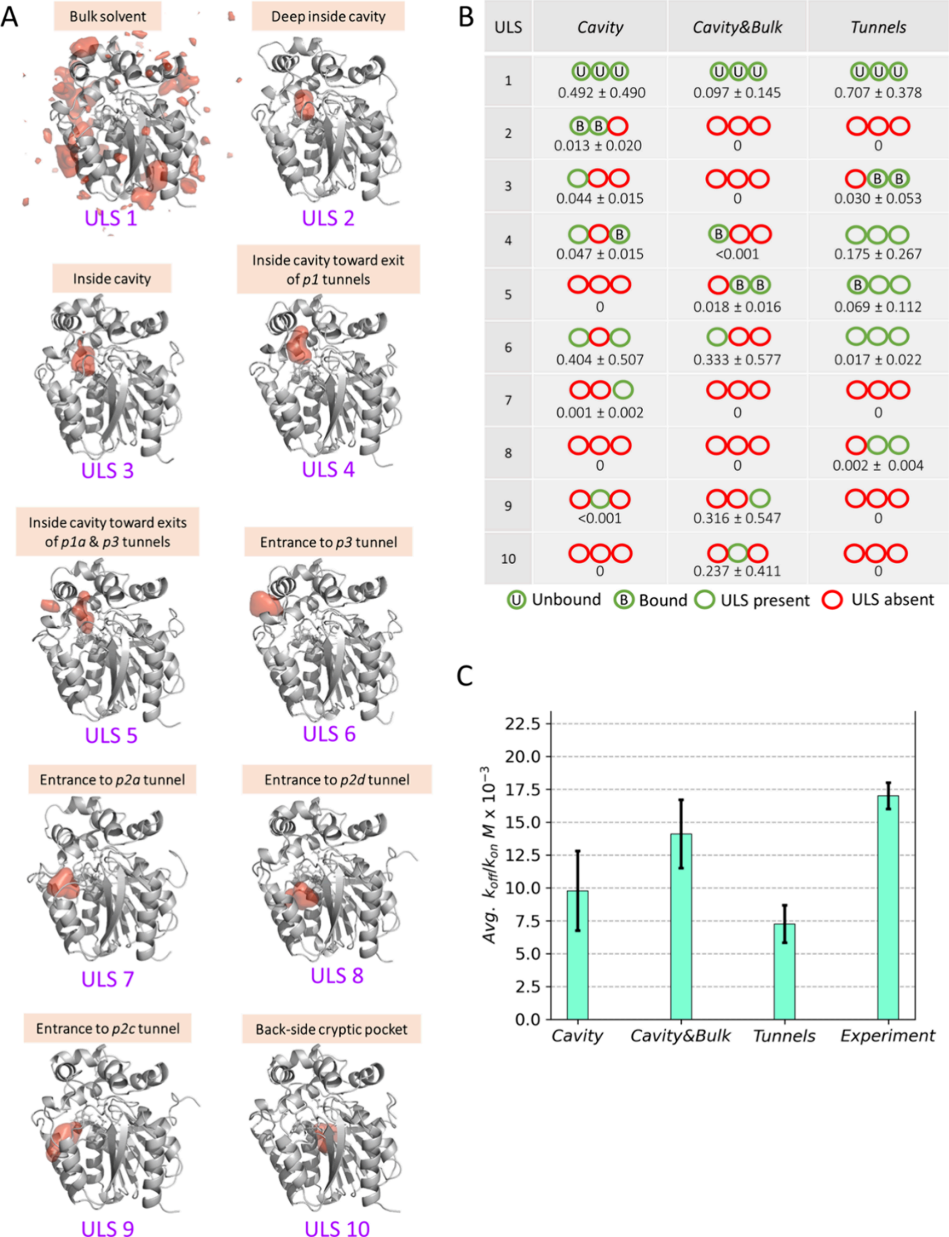
Inference about the (un)binding process of DBE to LinB86 from the MSM analysis. (A) Structurally unified ligand states (ULSs) identified among all metastable states (Figures S25–S33) resolved by MSM analysis of three replicated ASMD simulations initiated from the studied seeding schemes. Protein structures are shown as a gray cartoon, whereas the region occupied by the DBE molecule in 20% (1% for bulk solvent state) of 1,000 structures representing a given ULS is shown as a red surface. (B) Presence of ULS among metastable states in each ASMD replicates with their average probabilities. The unbound and bound metastable states used as source and sink states during the mean first-passage time analyses are highlighted (Table S4). (C) Average equilibrium dissociation constants derived from MSMs as the ratio of dissociation and association rates (Table S5). The experimental *k*_d_ was obtained from.^75^ The data represent mean ± s.e.m. from the three replicates.

Finally, these unified fingerprints enabled us to cluster the metastable states (Figure S38), forming unified nonredundant ligand states (ULSs) across all MSMs (Figure 4A). The only state consistently present in all replicates of each seeding scheme (Figure 4B) was ULS1, which corresponded to the DBE molecules in the bulk solvent. ULS2–ULS5 all represented metastable states of DBE molecules bound inside the active site cavity. The DBE was bound closest to the catalytic residues in ULS2, which was found only in the MSMs of the *Cavity* scheme. In ULS3, the substrate was placed closer to the cavity center, whereas in ULS4 and ULS5, the substrate was located near the exit from the cavity in the direction of the *p1* or *p3* tunnels. ULS6–ULS9 featured the DBE molecule bound on the LinB86 surface at the entrances to the *p3*, *p2a*, *p2c,* and *p2d* tunnels, with the *p3* tunnel entrance (ULS6) being the most prevalent across the MSMs (Figure 4B). Curiously, in replicate2 from the *Cavity&Bulk* scheme, we observed several metastable states forming ULS10, which were composed of DBE molecules exploring the cryptic pocket located back-to-back with the canonical active site cavity of LinB86 and with the entrance located on the opposite side of the enzyme structure with respect to the *p1* tunnel entrance.

Some identified ULSs were also observed in a recent study of transient binding sites on the LinB wild-type conducted with seven halogenated compounds, including DBE molecules.^72^ Out of nine sites, three could be matched to ULSs as follows: (i) site 5 corresponded to ULS6, the entrance to the *p3* tunnel, (ii) site 9 overlaid with ULS9, the entrance to the *p2c* tunnel, and (iii) site 4 aligned to ULS8, the entrance to the *p2d* tunnel. Such agreement suggests conservation of those interaction sites between LinB wild-type and LinB86 mutant despite the substitutions introduced into the *p1* and *p3* tunnels of the mutant. From the identification of ULSs in replicated MSMs, the *Tunnels* scheme exhibited the best consistency because four ULSs were found in all three replicates, whereas the other two ULSs were found in two replicates. In contrast, only unbound ULS1 was systematically found in the *Cavity* and *Cavity&Bulk* schemes. In fact, those two schemes frequently led to the formation of singleton ULSs, i.e., ULSs present in one replicate only.

Finally, using the metastable states with the largest population of DBE bound close to catalytic residues of the enzyme below 5 Å COM distance (Table S4) as the bound state (Figure 4B), we calculated the equilibrium dissociation constant (*k*_*d*_) from the rates of DBE association (*k*_on_) and dissociation (*k*_off_) predicted from MSMs by the mean first-passage time analyses. All three schemes exhibited comparable average association and dissociation rates for DBE (p-values >0.12), with the *Cavity&Bulk* scheme consistently having the lowest relative error. In contrast, the *Tunnels* scheme exhibited the largest relative errors for these rates that, however, exhibited partial cancelation when computing *k*_*d*_ values (Table S5). Interestingly, we found that the computed *k*_*d*_ values from all schemes agreed with the experimentally determined one within an order of magnitude (Figure 4C). However, only the *k*_*d*_ value obtained from the *Cavity&Bulk* scheme was not statistically different from the experimental data (Table S7). Considering the convergence of the computed *k*_*d*_, the *Cavity* scheme exhibited the largest relative error, surpassing 30% (Table S5). In general, it is still not common for such predicted data to reach this level of agreement with experiments, even for much less complex biomolecular systems.^73,74^

### 3.3 Exploration of Different Transport Paths by Substrate and Its Ability to Transit across Their Bottlenecks

Next, we investigated the utilization of individual transport pathways of LinB86 by the substrate DBE. Initially, we attempted to match the substrate migration traces to the tunnel ensembles using the TransportTools library.^76^ However, we observed very few complete migration events of the DBE molecule between the bulk solvent and the active site of LinB86 (Table S8), with replicate2 of the *Cavity* scheme capturing 33 transport events of DBE via known tunnels. Because such data did not allow sufficient inference, we considered a simplified transition of the DBE molecule through the tunnel bottleneck only, which corresponds to the least favorable region along the migration path and, hence, controls the transport rates.^9,69,75^ By considering the distances of DBE to the COM of bottleneck residues of each tunnel and the bottom of the active site cavity (Figure S22), we traced the location of DBE in all simulations, focusing on the frames where DBE came close to any of the bottlenecks and whether it passed through them.

**Figure 5:**
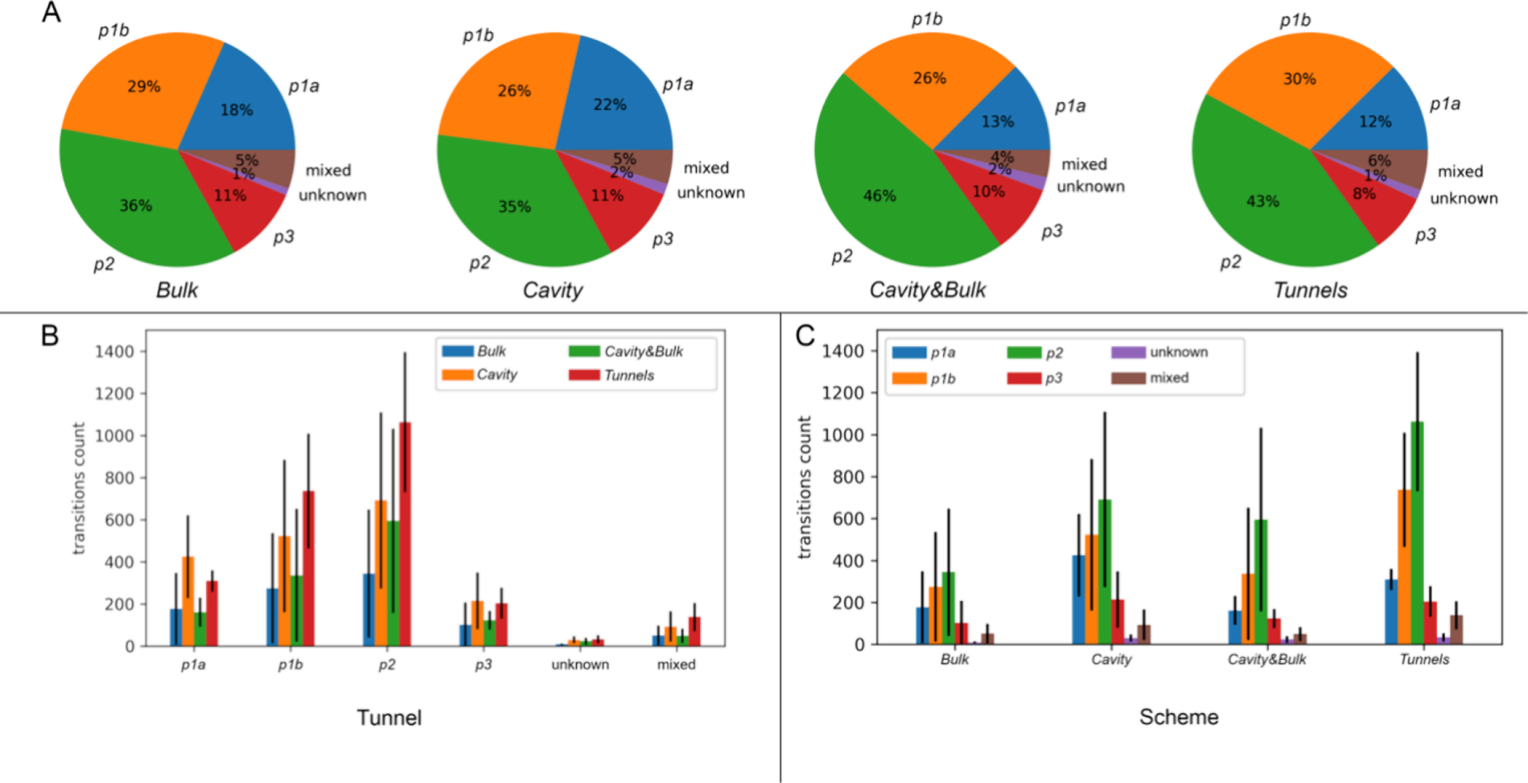
Tunnel utilization by the DBE in the investigated seeding schemes. (A) Relative utilization of particular tunnels for each scheme. (B) Per tunnel average tunnel utilization. (C) Per scheme average tunnel utilization. The data in B and C represent mean ± stdev from the three replicates.

A thorough investigation of the transport in all schemes via particular tunnels revealed the following observations. We observed the highest total number of transitions for the *Tunnels* scheme, followed by *Cavity*, *Cavity&Bulk*, and finally, the lowest for the *Bulk* only (Table S9). The overall proportion of the particular tunnels utilized to the total number of transitions was consistent across all schemes. The most frequently used tunnel was *p2*, followed by *p1b*, *p1a*, and *p3,* whereas mixed and unknown transport routes represented the smallest fraction of the data (Figure 5A). We have visually explored the nature of unknown transitions to verify if they correspond to the usage of a new tunnel. These transitions were primarily located in the vicinity of known tunnels but beyond the used cutoff values. However, in replicate2 of *Cavity&Bulk* scheme, we have also observed transitions of DBE into the back-side cryptic pocket, i.e., ULS10 among the unknown ones (Figure 4A).

Interestingly, besides this consistent trend in tunnel usage, schemes *Cavity&Bulk* and *Tunnels* displayed a higher percentage of the *p2* tunnel than the remaining schemes (Figure 5A), suggesting that the more complex seeding schemes enabled relatively more efficient exploration of the longest and most complex branches of the *p2* tunnel. In contrast, simplified schemes (*Bulk* and *Cavity*) tended to promote the sampling of more accessible primary conduits (i.e., the *p1* tunnel) in agreement with their preferential utilization observed in TransportTools analyses of replicate2 of the *Cavity* scheme (Table S8). Besides this difference, the increased number of transitions for particular schemes mainly came from the proportionally boosted sampling for each tunnel (Figure 5B). Importantly, given the standard deviations of the three replicates for each scheme, it is clear that the *Tunnels* scheme had the highest consistency of all tested schemes for all tunnels. This was particularly evident when the transitions were considered for each run separately (Table S9). Whereas the total sum of transitions for the *Bulk* scheme differed noticeably for particular replicates (1820, 49, and 1006 transitions for replicate1, replicate2, and replicate3, respectively), the *Tunnels* scheme had the lowest deviation when the three replicates were considered separately (replicate1–2809, replicate2–1680, replicate3–2968). *Cavity* and *Cavity&Bulk* schemes fell between those two extremes and had a similar consistency to each other (Figure 5C).

### 3.4 Lessons Learned

By contrasting the performance of individual seeding schemes from different perspectives, we could identify several benefits and limitations. First, by infusing more knowledge into the initial seeds, we can drive ASMDs to sample relevant protein–ligand space, enabling appreciable agreement with the experiment (Figure 4C). However, in the case of the *Cavity* scheme, this was possible by virtue of the investigated LinB86 that does not bind the DBE substrate too tightly and hence allows exploration of the unbinding process well enough. As such, we would not expect that this scheme would perform well when applied to cases like the trypsin–benzamidine system.^33,35^ The more knowledge-based schemes, especially *Tunnels*, exhibited notably higher reproducibility and faster convergence in the proportion of explored regions (Figures 2, 3, 4C, and Table S5). This consistency indicates that even ASMD setups with fewer epochs might provide interesting insights into the transport processes without requiring intensive computational efforts. For illustration, a 45 μs ASMD run consisting of 30 epochs with 900 simulations, representing a typical use-case for investigating similar systems, required circa 9 days of computations on 18 RTX2080 cards.

From the perspective of studying the utilization of multiple tunnels present in the LinB86, the *Tunnels* schemes clearly promoted in-depth exploration of tunnel region (Figures 2, 3, and 5), providing the most complete overview of tunnel usage by DBE. Therefore, the *Tunnels* scheme would be particularly beneficial when investigating proteins with complex tunnel networks. Here, we noted a clear drawback of the exploration preference of ASMDs when information-rich and hard-to-explore tunnel networks are initially seeded, is that it resulted in a quite limited sampling of the active site and bulk regions, indicating that more hybrid seeding of tunnels with some seeds being placed in the active site cavity as well as in bulk regions could yield more balanced sampling.

Despite its rather beneficial properties, ASMDs using *Tunnels* seeding generally suffer from limitations originating from its dependence on tunnel detection in preseeding simulation(s) (Text S1). The placement of ligands to the originally identified tunnels will prioritize their sampling, and only after an active site or unbound regions are visited will the ASMD progress to explore these regions, including other potentially overlooked tunnels. Hence, the discovery of other tunnels might be delayed due to thorough exploration of the seeded ones. This limitation could, to some extent, be overcome by analyzing the tunnels with a rather small probe, as small as 0.7 Å, which was shown to enable the identification of even potential tunnels that could be formed after gain-of-function mutations or induced by ligand binding.^10,77^ In this study, we have used a probe radius of 0.9 Å, which sufficiently well identified the known tunnels of Linb86. However, even <0.7 Å probe could readily be applied without incurring extreme computational costs by using the Divide-and-Conquer approach.^78^

The applied seed generation based on rather short standard MD simulation(s) can be expected to capture relevant tunnels that are gated primarily with side chains but will be less appropriate for proteins requiring pronounced conformational changes to facilitate tunnel opening and ligand migration. While the molecular gates formed only by side-chains are the most prevalent,^9,10^ for systems exhibiting larger transformations, the tunnels could be identified using enhanced sampling methods, like Gaussian accelerated MD simulations, which was shown to be applicable for this purpose.^79^ Along the same line, moderately sized ligands would most probably fit into some of the tunnel instances of the ensemble generated by apo simulations performed here. However, larger ligands would require either seeding with the *Cavity&Bulk* scheme or their explicit inclusion in simulations, using computationally demanding biasing methods capable of enforcing the induction in the tunnel geometry due to the ligand presence, akin to IterTunnel approach,^80^ which, however, could result in the formation of unrealistic tunnels when incorrectly applied.^81^

Finally, as ASMD does not utilize any form of energy biasing, no matter the seeding scheme used, there will certainly be protein–ligand systems presenting too large energy barriers to witness ligand transitions with underlying standard MD simulation protocols despite significant computational efforts. In those instances, the *Tunnels* seeding scheme could be employed to obtain input structures for enhanced sampling methods, like umbrella sampling,^82^ which could be used to sample these high-energy regions and later integrated via multiensemble MSM, to attain a comprehensive view of ligand migration in the given protein system.^38^

## 4 Conclusions

This study aimed to test the effect of different seeding schemes on the sampling of metastable states of LinB86-DBE complexes in MSM-driven ASMD simulations. Meaningful insights were provided into the kinetic rates and mechanisms of the transport of the substrate DBE in LinB86 from its deeply buried active site to the solvent environment via multiple transport tunnels. Four designed seeding schemes were used to position DBE by using more knowledge to tackle the sampling of regions with higher energy barriers. The ensuing ASMD simulations constructed kinetic models with different levels of detail based on each employed seeding scheme. All simulations explored the entire transport process, visiting unbound and bound states except for the *Bulk* scheme, which could not reach the bound state in two replicates of 45-μs ASMDs. Conversely, the *Tunnels* scheme was the most consistent in sampling different metastable states of the substrate in the transport-relevant regions. Application of the more information-rich *Tunnels* and *Cavity&Bulk* schemes led to the enhanced exploration of the auxiliary *p2* and *p3* tunnels. In contrast, the primary *p1* tunnel was preferred in ASMDs initiated from the other two schemes. *Tunnels* and *Cavity&Bulk* schemes also provided the most converged *k*_*d*_ values from the rates of DBE association and dissociation, which were sufficiently close to the experimental measurements (within an order of magnitude) despite the complexity of the kinetic models. We expect that a methodology analogous to the one used during the seeding of *Tunnels* scheme (Text S1) could be beneficial for the identification of relevant protein–ligand states along such a likely migration path and subsequently defining suitable collective variables for enhanced sampling methods targeting ligand migrations, such as metadynamics^21,22^ and umbrella sampling.^82^ Overall, the infusion of more knowledge into the initial seeds of ASMD simulations could render computational analyses of transport mechanisms in enzymes more consistent, even for very complex biomolecular systems. This has a clear potential to translate into faster rational protein design and drug development efforts.

## Data Availability

The underlying data for this study are available in the published article, the Supporting Information, and Zenodo repository at 10.5281/zenodo.10849386. The data include Python scripts, binary files, plain text, and PDB-formatted and AMBER-formatted structural data, all compatible with various freely available SW packages. No tools with restricted access are required.

## Supporting Information Available

Properties of tunnel identified in LinB86 simulation. localization of DBE molecule around the LinB86 enzyme used as initial seeds in four investigated schemes. Protocol describing MD simulation used for tunnel calculations during seeding with scheme *Tunnels.* DBE migration energy barriers profile of ensembles of investigated tunnels. Scheme showing the creation of composite tunnel used for DBE seeding. Implied time scales of generated MSM, corresponding spectral separation analysis, and Chapman-Kolmogorov tests for all replicates of *Cavity*, *Cavity**&Bulk*, and *Tunnels* schemes. Visualization of locations of the COM of residues used to characterize DBE locations within the LinB86 structure and their RMSFs for all schemes replicates. Statistics on the successfully completed ASMDs for all schemes replicates. Protein RMSD and RMSF calculated from ASMD simulations for all schemes replicates. Visualization of region preferentially occupied by DBE molecule at approximately 25 Å-distance from the catalytic residues. Visualization of metastable states and characteristic distances of their representative structures for all replicates of *Cavity*, *Cavity**&Bulk*, and *Tunnels* schemes. Clustering of metastable states from all MSM analyses into ULS. Association and dissociation rates derived from MSMs for *Cavity*, *Cavity**&Bulk*, and *Tunnels* schemes. Configurations in metastable states with DBE within 5 Å from the catalytic residues. Kinetic rates of DBE with LinB86 derived from MSMs. Sensitivity of calculated *k*_off_/*k*_on_ values to the bound state selection. Statistical comparison between experimental and calculated *k*_d_ values. Number of substrate migration events obtained from TransportTools analyses for all replicates of *Cavity*, *Cavity**&Bulk*, and *Tunnels* schemes. Statistics of bottlenecks’ crossing by DBE molecule for all schemes replicates (PDF)
