# Supplementary material for: Incorporating Prior Knowledge in the Seeds of Adaptive Sampling Molecular Dynamics Simulations of Ligand Transport in Enzymes with Buried Active Sites
Source: J Chem Theory Comput. 2024 Jul 9;20(14):5807–19. doi: 10.1021/acs.jctc.4c00452 (PMC11270739; doi:10.1021/acs.jctc.4c00452)
Supplement: Supplementary file 1 — ct4c00452_si_001.pdf [file ct4c00452_si_001.pdf]

Supporting Information

Incorporating prior knowledge in the seeds of adaptive sampling molecular dynamics simulations of ligand transport in enzymes with buried active sites

Dheeraj Kumar Sarkar,[1,2] Bartlomiej Surpeta,[1,2] Jan Brezovsky*[1,2]

[1] Laboratory of Biomolecular Interactions and Transport, Department of Gene Expression, Institute of Molecular Biology and Biotechnology, Faculty of Biology, Adam Mickiewicz University, Uniwersytetu Poznanskiego 6, 61-614 Poznan, Poland.

[2] International Institute of Molecular and Cell Biology in Warsaw, Ks Trojdena 4, 02-109 Warsaw, Poland.

*Corresponding author: JB: janbre@amu.edu.pl

Table S1. Properties of tunnel identified in LinB86 simulation using CAVER.

ID	*No*	*No_snaps*	*Avg_BR*	*SD*	*Max_BR*	*Avg_L*	*SD*	*Avg_C*	*SD*	*Priority*	*Avg_throughput*	*SD*
p1a	121	121	1.1	0.2	1.6	16.0	2.5	1.4	0.2	0.11	0.46	0.09
p1b	146	146	1.3	0.2	1.8	13.6	1.7	1.3	0.1	0.17	0.59	0.10
p2a	231	231	1.2	0.2	1.8	20.0	2.5	1.3	0.1	0.22	0.47	0.10
p2c	237	237	1.0	0.1	1.6	22.9	2.7	1.4	0.2	0.18	0.37	0.06
p2b	184	184	1.1	0.2	1.6	17.1	2.4	1.4	0.2	0.17	0.47	0.08
p2d	164	164	1.0	0.1	1.5	25.6	3.1	1.8	0.3	0.11	0.33	0.07
p3	97	97	1.0	0.1	1.4	16.3	2.0	1.3	0.1	0.09	0.45	0.07

**ID: tunnel cluster ranked based on priority, No: Total number of tunnels, No_snaps: Number of snapshots, Avg_BR: Average bottleneck radius (Å), Max_BR: Maximum bottleneck radius (Å), Avg_L: Average tunnel length (Å), Avg_C: Average tunnel curvature, Priority: Tunnel priority calculated by averaging tunnel throughputs over all snapshots, Avg_throughput: Average tunnel throughput.*

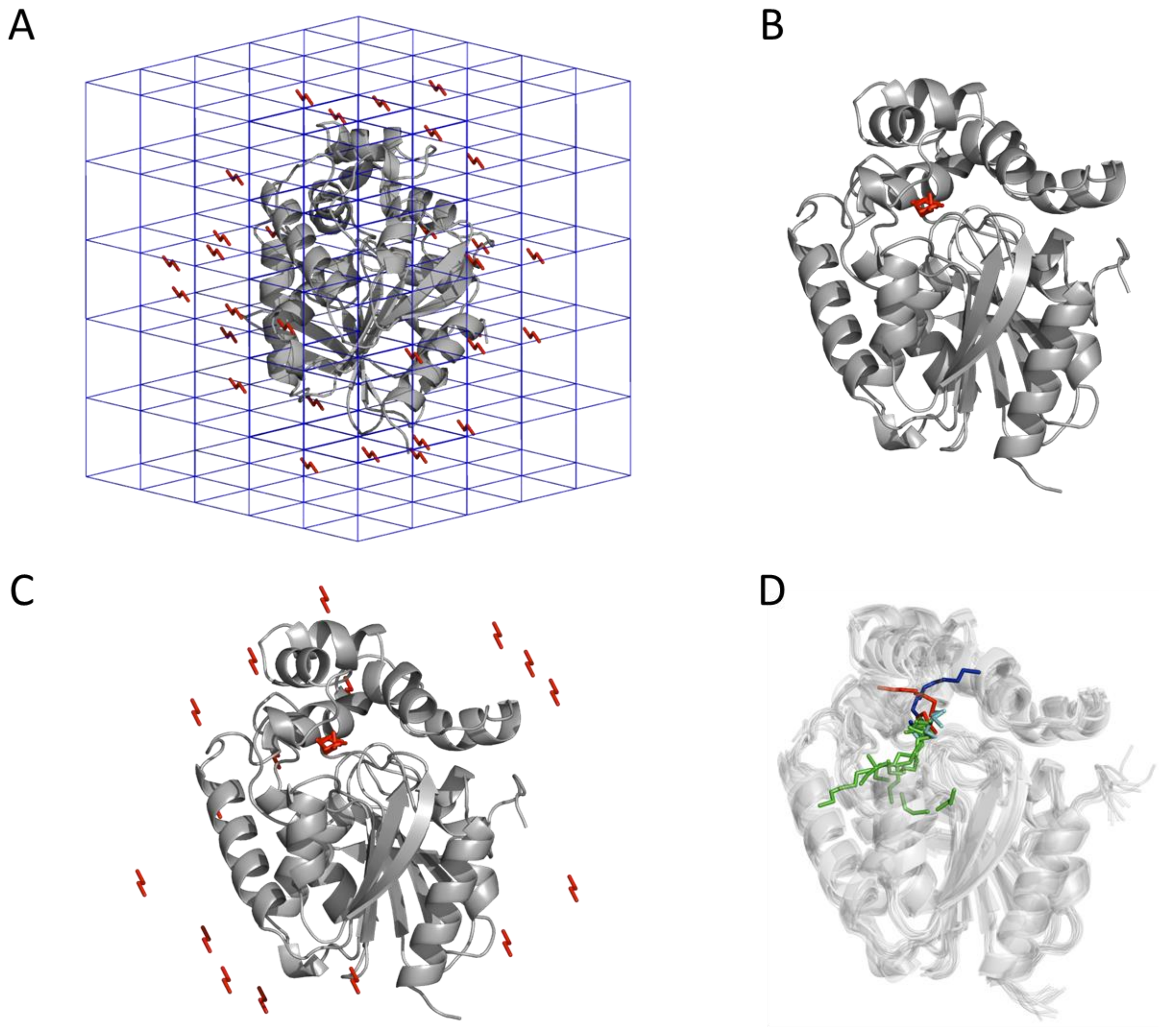

Figure S1. Initial seeds of the DBE molecule around the LinB86 enzyme used in four investigated schemes. A) *Bulk*, B) *Cavity*, C) *Cavity&Bulk,* and D) *Tunnels* schemes. In the *Tunnels* scheme, not only positions of DBE molecules were seeded, but also the corresponding structure of LinB86 harboring appropriate tunnel fragments was used. Protein structure is shown as gray (static) or white (ensemble) cartoon. Positions of DBE are shown in red sticks, except for the *Tunnels* scheme in which sticks are colored according to the tunnel seeded: *p1a* (blue), *p1b* (cyan), *p2* (green), and *p3* (red).

Text S1 Protocol for MD simulation for tunnel calculations used during seeding with scheme *Tunnels:* For MD setup, AMBER18[1] package was used and the crystal structure of LinB86 was retrieved from the PDB database (ID 5LKA). The periodic boundary conditions were maintained by particle mesh Ewald method[2] with the SHAKE algorithm for 4 fs time-step and hydrogen mass repartitioning method[3]. For minimization, the system was subjected to five rounds of minimization consisting of 500 steps of steepest descent minimization. After minimization, the system was gradually heated at canonical NVT ensemble from 0 K to 200 K with restrained heavy protein atoms for 20 ps using the Langevin thermostat. Then the system was equilibrated for 2 ns in three rounds. First using harmonic restraints, the system was gradually heated to 310 K within 100 ps and keeping constant temperature for another 900 ps in canonical NVT ensemble. Secondly, the system was subjected to constant pressure and temperature (NPT) while restraining the backbone atoms for 1 ns, using a weak-coupling barostat. Finally, 100 ns of NPT simulation without restraints was performed.

The MD trajectory was analyzed to identify geometric tunnels by CAVER 3.0.1[4,5] with the starting points of tunnels defined by the following active site residues: Asn38, Asp108, Trp109, and His272, using a probe radius of 0.9 Å, shell radius 3 Å and shell depth 4 Å. The obtained tunnels were clustered by hierarchical clustering with a clustering threshold of 3.5 Å. For each known tunnel, i.e., *p1a*, *p1b*, *p2,* and *p3* (Table S1), the 100 most opened tunnels were selected for positioning the DBE molecule along the entire tunnel length by the CaverDock program[6]. As a result, sets of DBE positions along the tunnel and the corresponding upper-bound interaction energies were generated for each tunnel ensemble. These energies were converted to approximate maximal migration barriers for tunnel segments of 1 Å length by finding the highest energy in the segment and subtracting the global minimal interaction energy found among the entire tunnel ensemble (Figure S2-S8).

Next, we have generated composite tunnels with putatively minimal energy costs for DBE migration through the respective tunnel conformations (Figure S9). For each tunnel segment, the tunnel conformation with the lowest migration barriers for DBE transport was selected unless the barrier of the previous optimal tunnel conformation was within 1 kcal/mol for this segment too. In such a case, the same tunnel conformation was retained to avoid frequent switching between the tunnel conformations. This procedure resulted in the generation of a subset of tunnel segments with the corresponding protein conformations with favorably bound DBE along the whole tunnel length (Figure S1D).

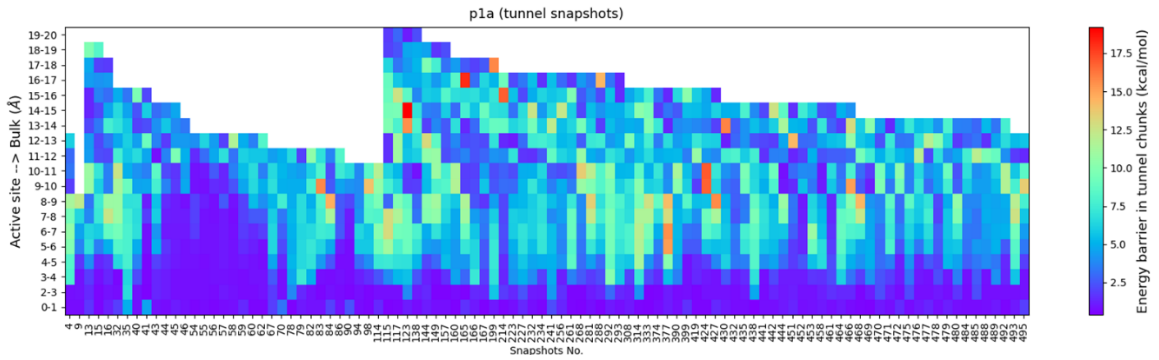

Figure S2. DBE migration energy barriers profile of *p1a* tunnel ensemble. The values correspond to the maximal interaction energy in each bin (1 Å length) predicted by CaverDock decreased by the global minimal interaction energy in the tunnel ensemble (-3.1 kcal/mol).

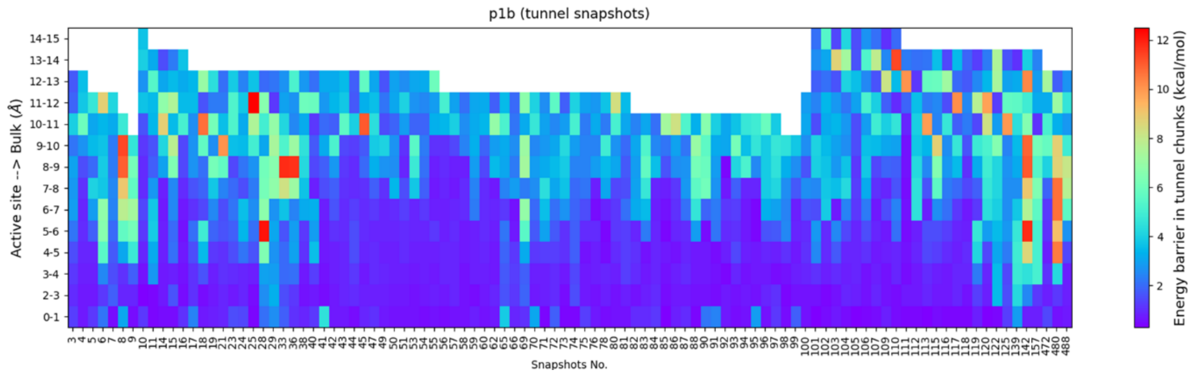

Figure S3. DBE migration energy barriers profile of *p1b* tunnel ensemble. The values correspond to the maximal interaction energy in each bin (1 Å length) predicted by CaverDock decreased by the global minimal interaction energy in the tunnel ensemble (-3.0 kcal/mol).

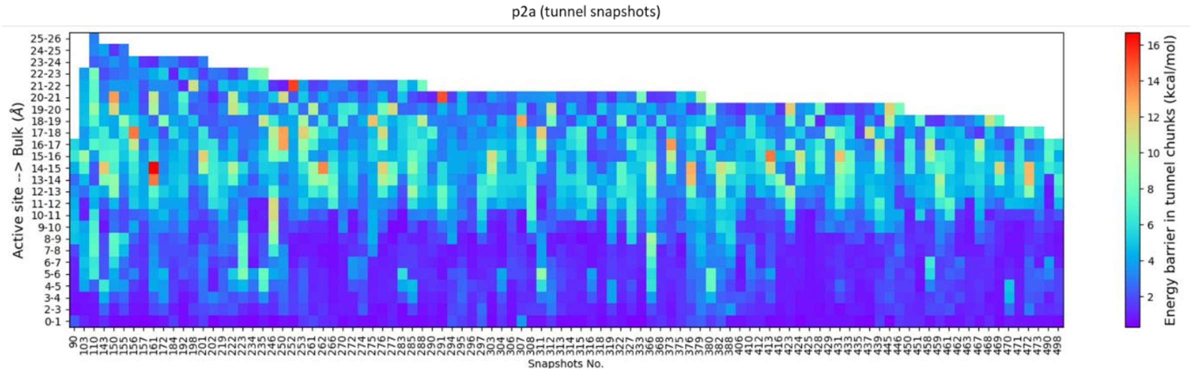

Figure S4. DBE migration energy barriers profile of *p2a* tunnel ensemble. The values correspond to the maximal interaction energy in each bin (1 Å length) predicted by CaverDock decreased by the global minimal interaction energy in the tunnel ensemble (-3.0 kcal/mol).

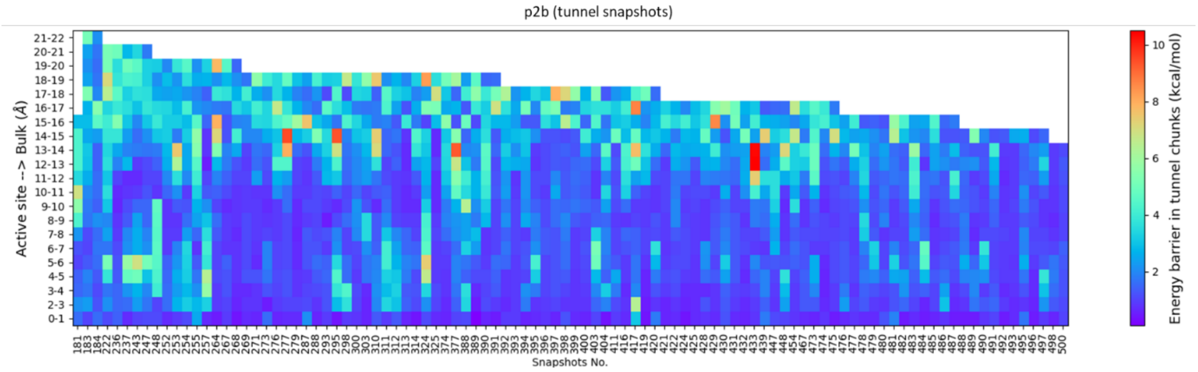

Figure S5. DBE migration energy barriers profile of *p2b* tunnel ensemble. The values correspond to the maximal interaction energy in each bin (1 Å length) predicted by CaverDock decreased by the global minimal interaction energy in the tunnel ensemble (-3.0 kcal/mol).

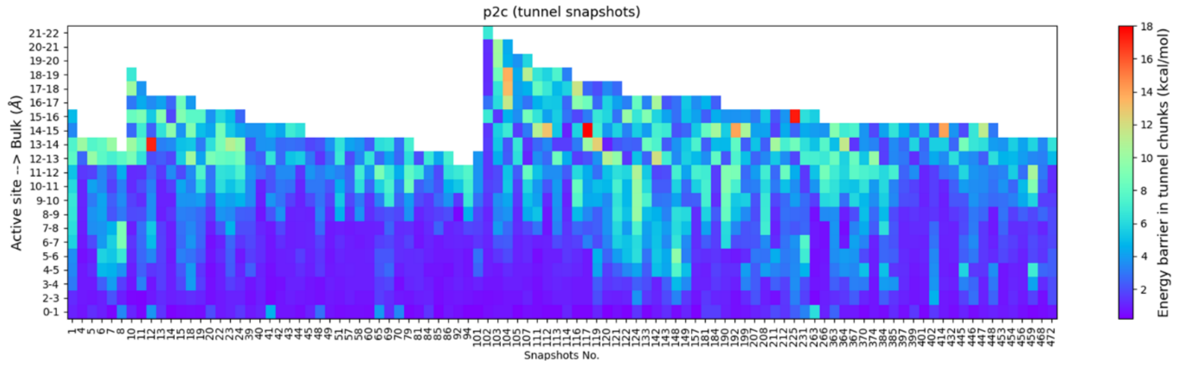

Figure S6. DBE migration energy barriers profile of *p2c* tunnel ensemble. The values correspond to the maximal interaction energy in each bin (1 Å length) predicted by CaverDock decreased by the global minimal interaction energy in the tunnel ensemble (-3.0 kcal/mol).

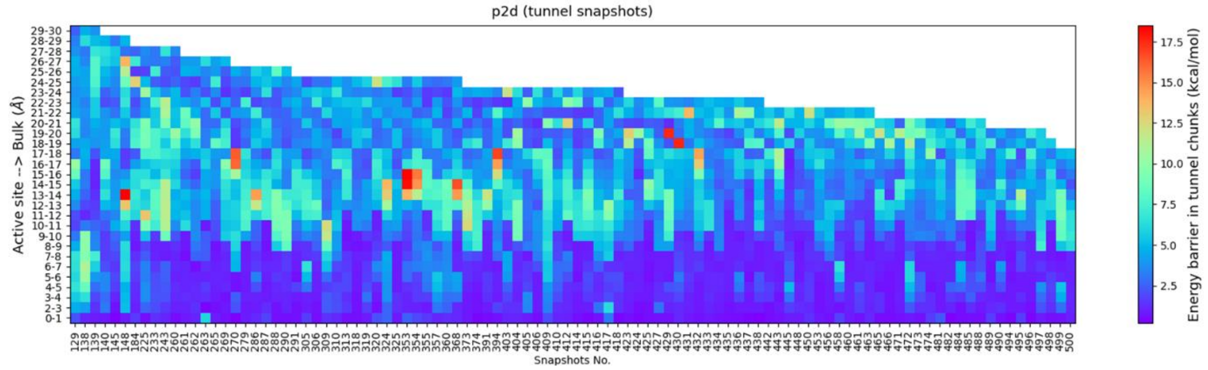

Figure S7. DBE migration energy barriers profile of *p2d* tunnel ensemble. The values correspond to the maximal interaction energy in each bin (1 Å length) predicted by CaverDock decreased by the global minimal interaction energy in the tunnel ensemble (-3.0 kcal/mol).

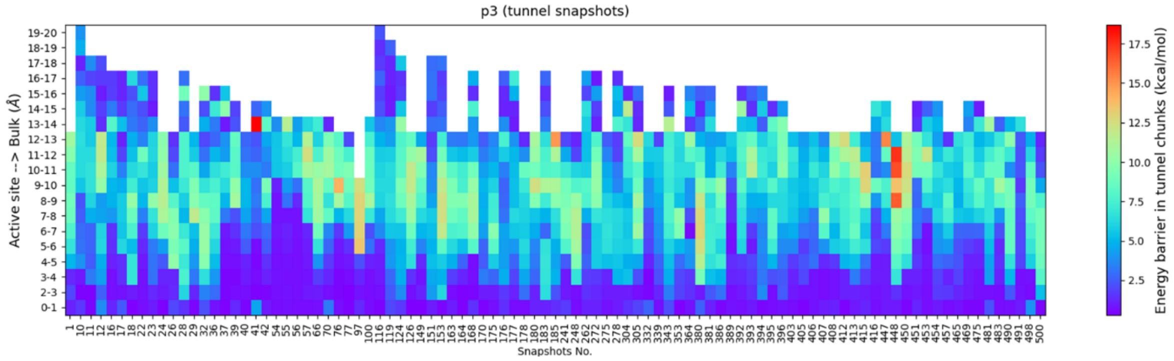

Figure S8. DBE migration energy barriers profile of *p3* tunnel ensemble. The values correspond to the maximal interaction energy in each bin (1 Å length) predicted by CaverDock decreased by the global minimal interaction energy in the tunnel ensemble (-3.0 kcal/mol).

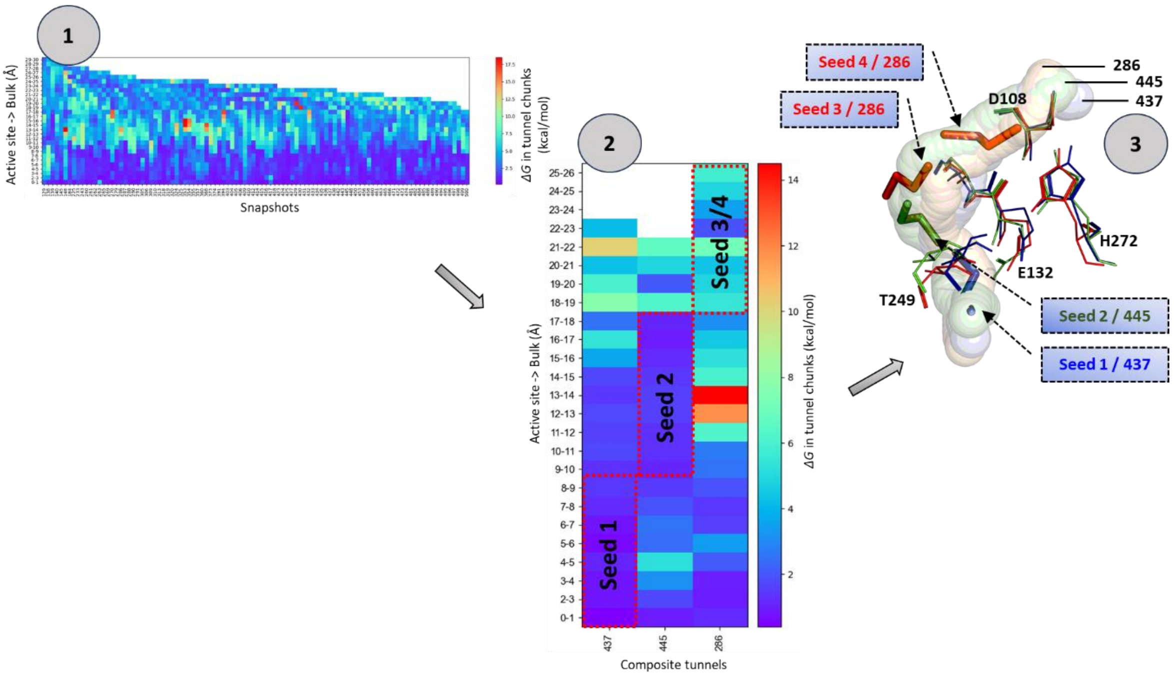

Figure S9. Creation of composite tunnel with corresponding bound poses of DBE molecule for seeding. *Step 1:* Compiling the ensemble of tunnels and maximal migration barriers for DBE molecule computed based on CaverDock energy profiles for each tunnel. *Step 2:* generation of composite tunnels (from several favorable segments of tunnels from the ensemble). Finally, in *Step 3*: the positions of ligands are selected for seeding based on the composite tunnel profiles by placing the DBE in favorable positions.

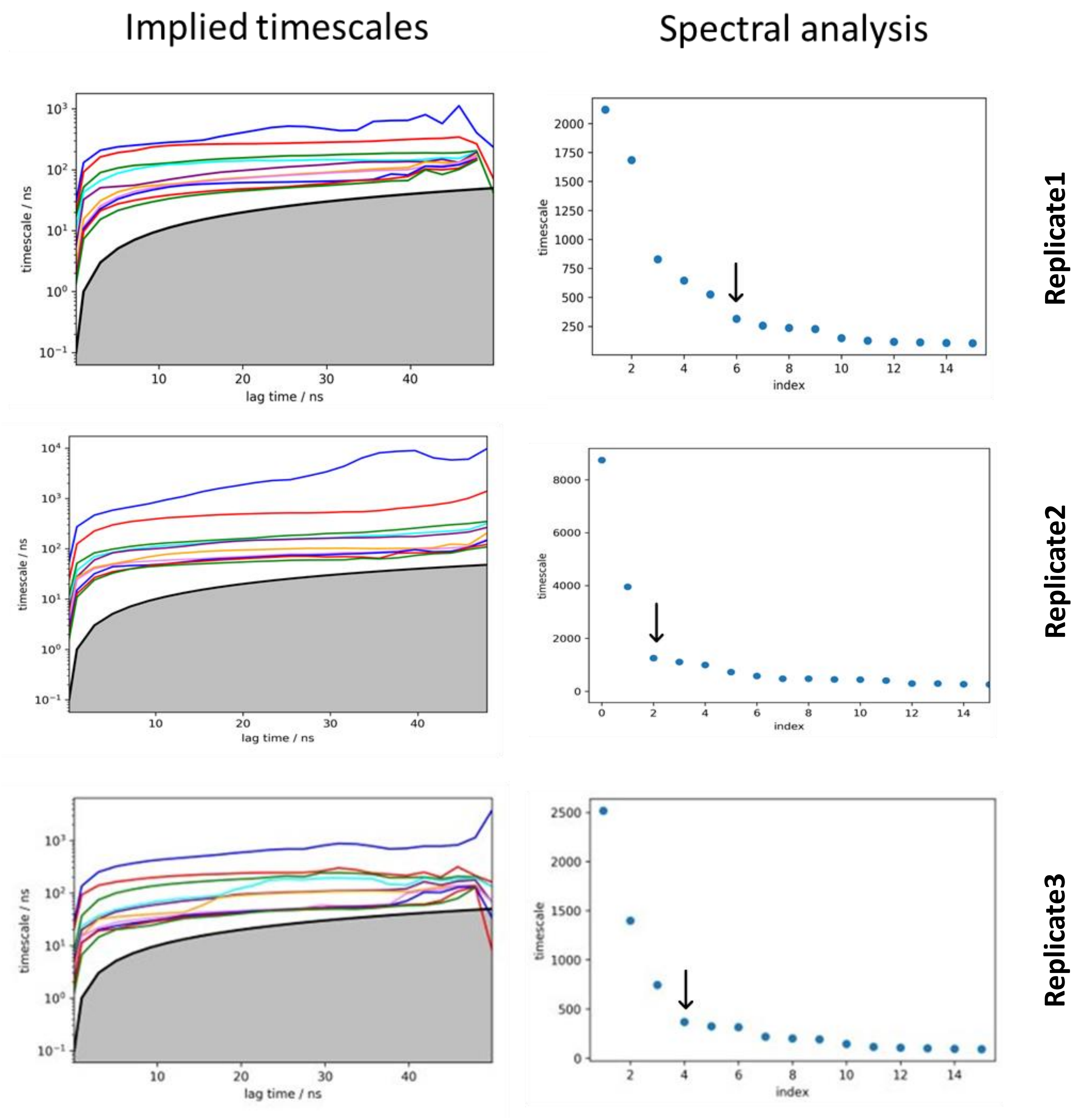

Figure S10. Implied time scales of MSM generated for three replicates of the *Cavity* scheme and corresponding spectral separation analysis. The arrow indicates the last point considered for deciding the number of metastable states.

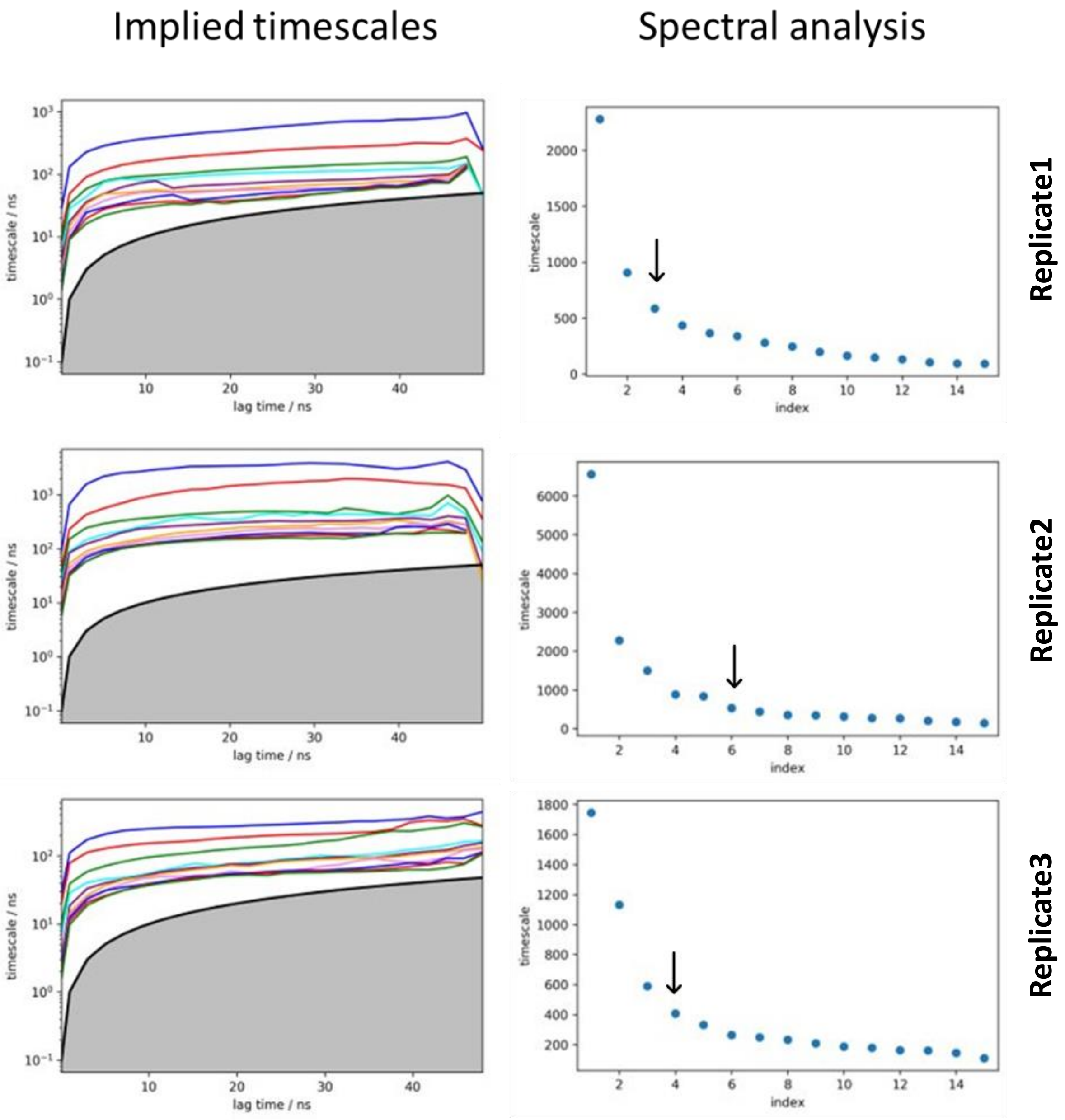

Figure S11. Implied time scales of MSM generated for three replicates of the *Cavity&Bulk* scheme and corresponding spectral separation analysis. The arrow indicates the last point considered for deciding the number of metastable states.

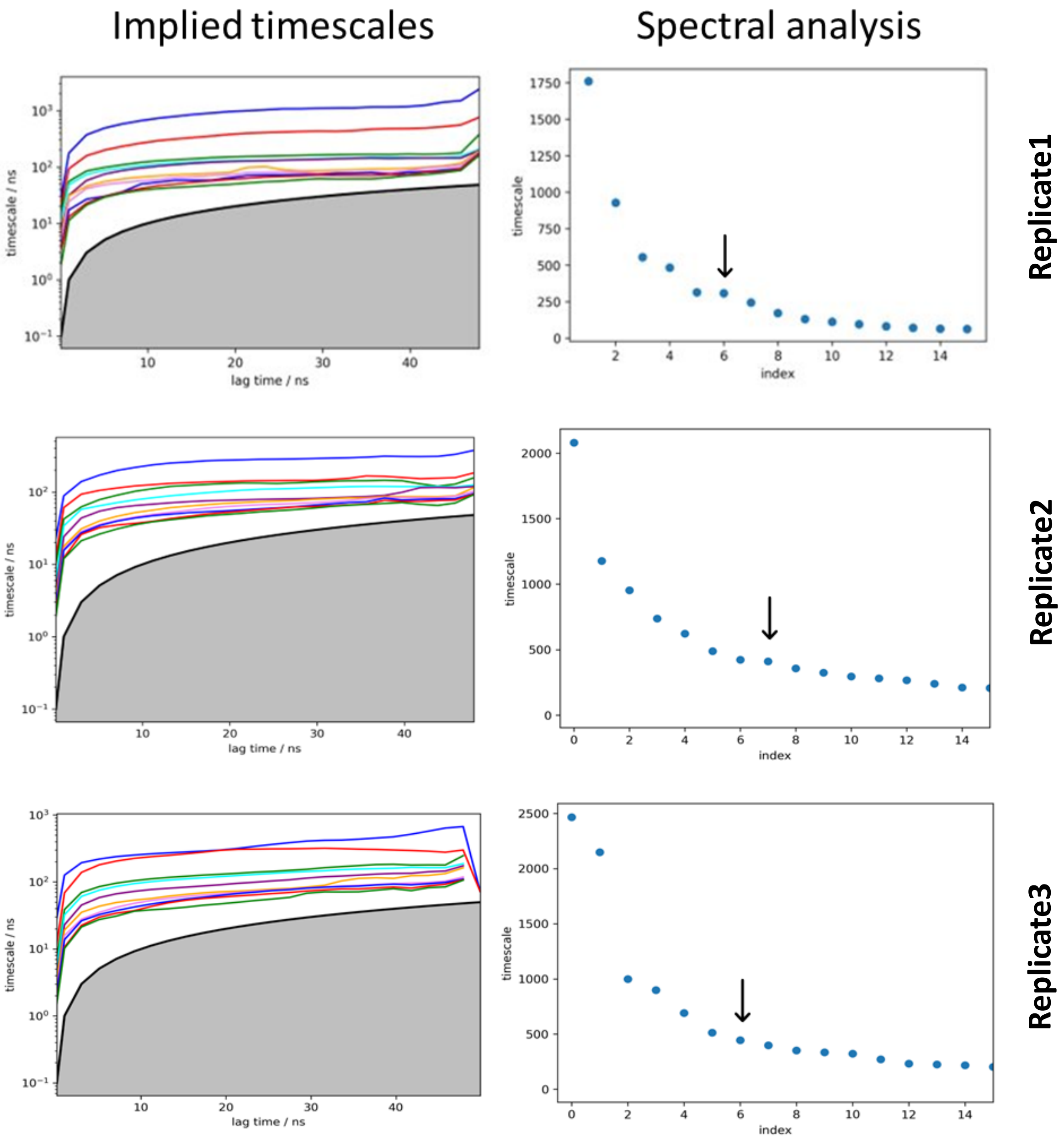

Figure S12. Implied time scales of MSM generated for three replicates of the *Tunnels* scheme and corresponding spectral separation analysis. The arrow indicates the last point considered for deciding the number of metastable states.

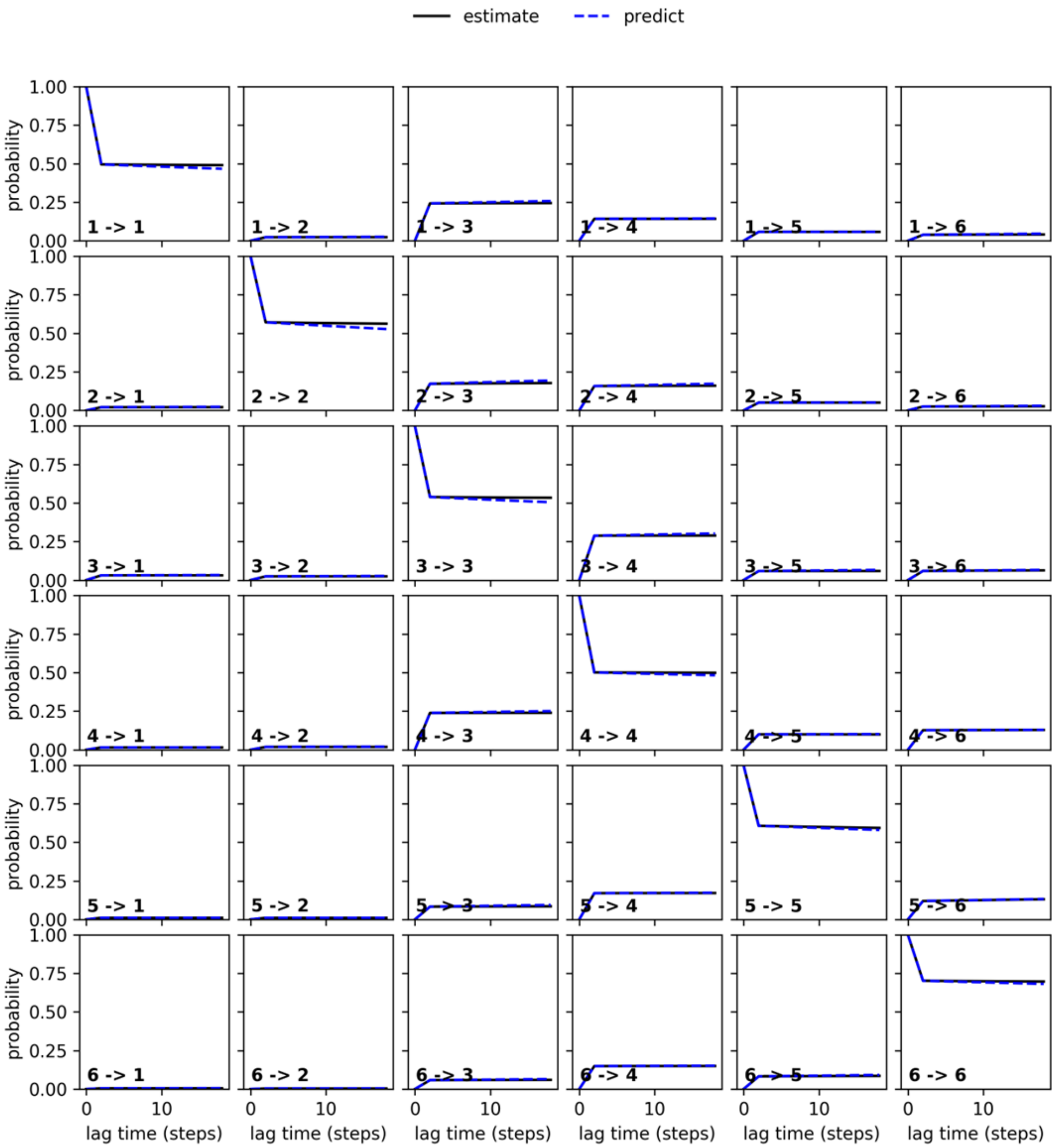

Figure S13. Chapman-Kolmogorov tests for replicate 1 of the *Cavity* scheme. Plots show that MSMs correctly represent original MD simulation data.

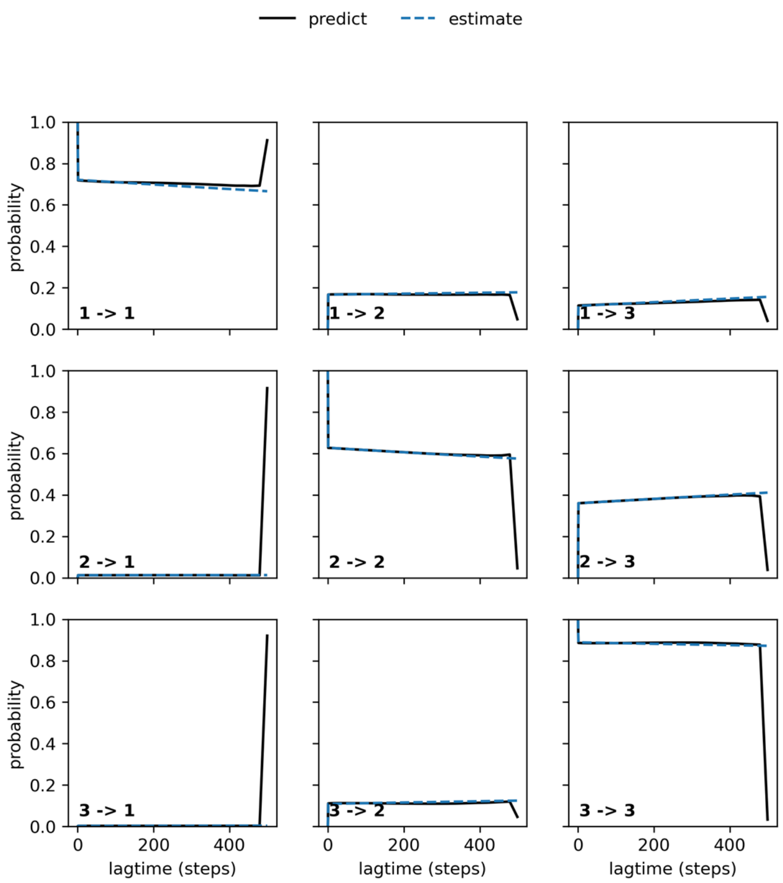

Figure S14. Chapman-Kolmogorov tests for replicate 2 of the *Cavity* scheme. Plots show that MSMs correctly represent original MD simulation data.

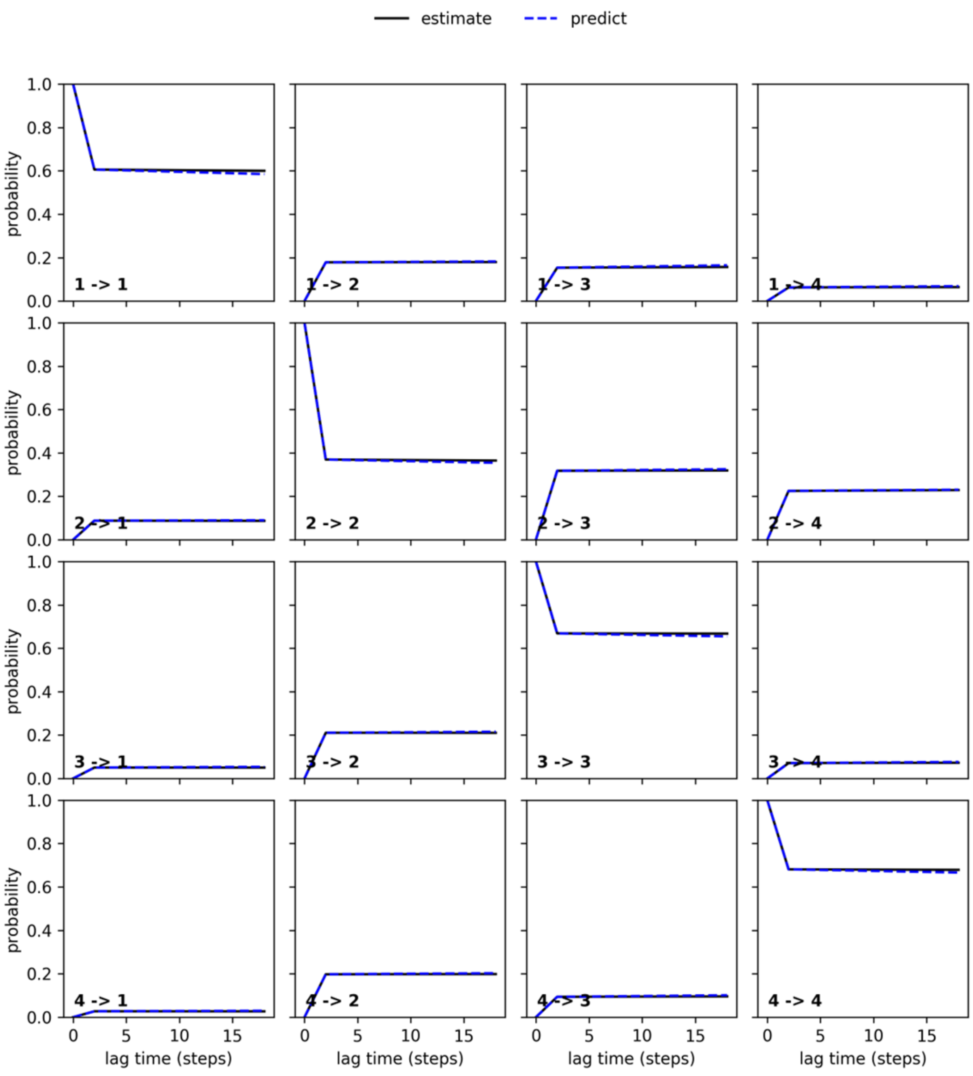

Figure S15. Chapman-Kolmogorov tests for replicate 3 of the *Cavity* scheme. Plots show that MSMs correctly represent original MD simulation data.

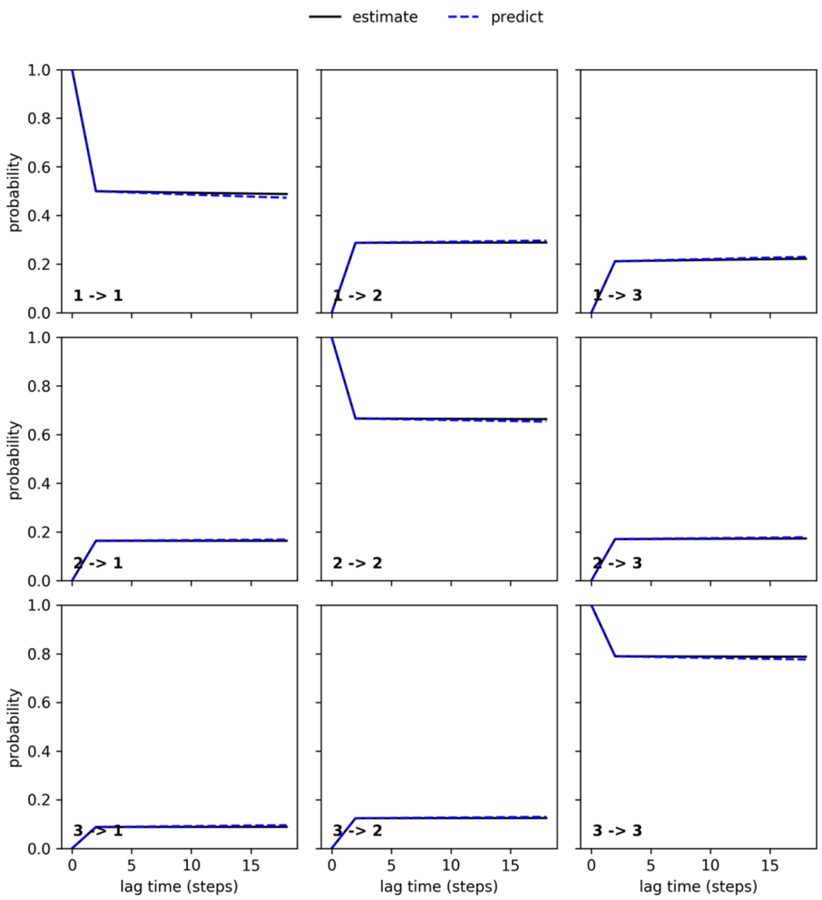

Figure S16. Chapman-Kolmogorov tests for replicate 1 of the *Cavity&Bulk* scheme. Plots show that MSMs correctly represent original MD simulation data.

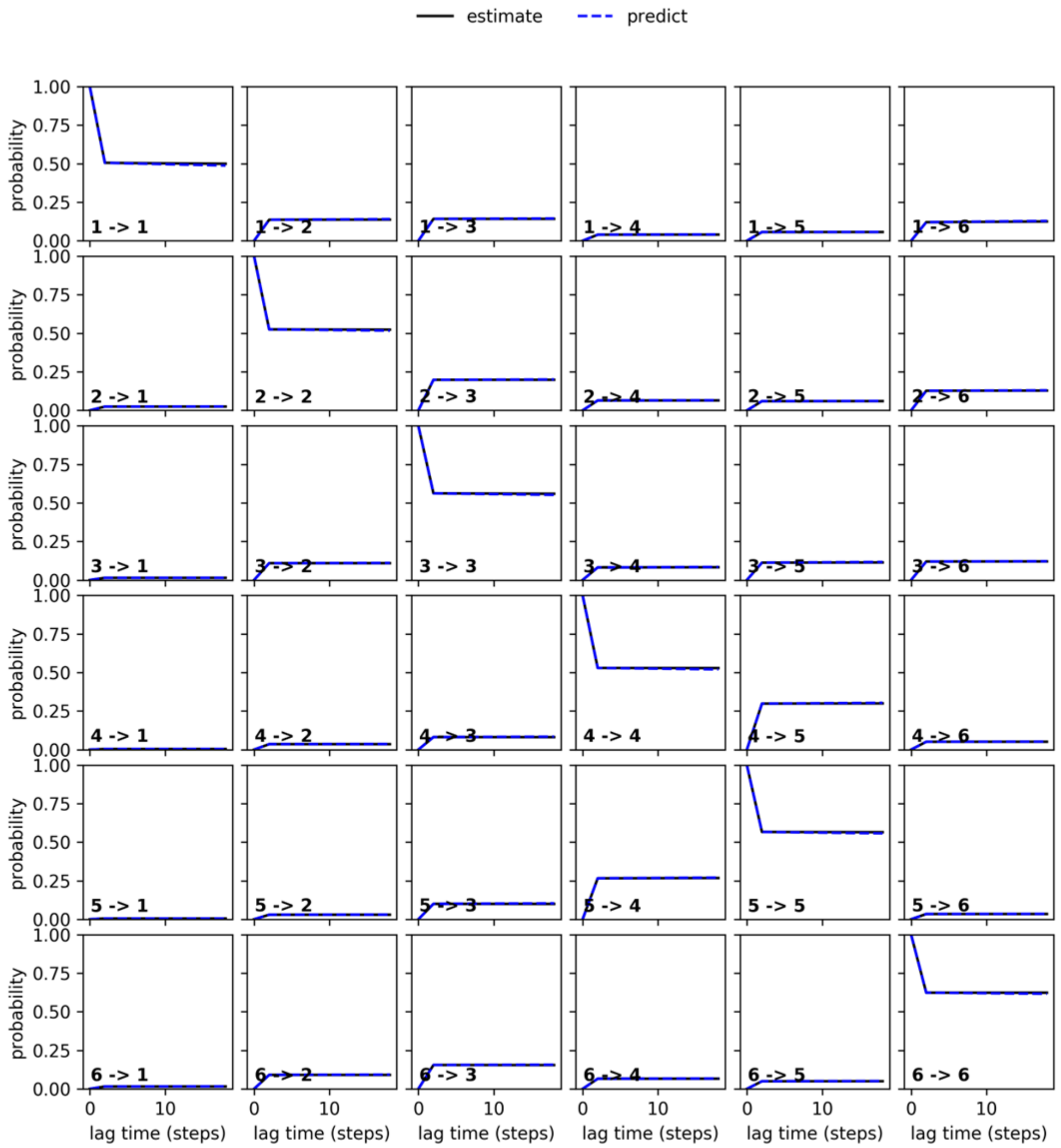

Figure S17. Chapman-Kolmogorov tests for replicate 2 of the *Cavity&Bulk* scheme. Plots show that MSMs correctly represent original MD simulation data.

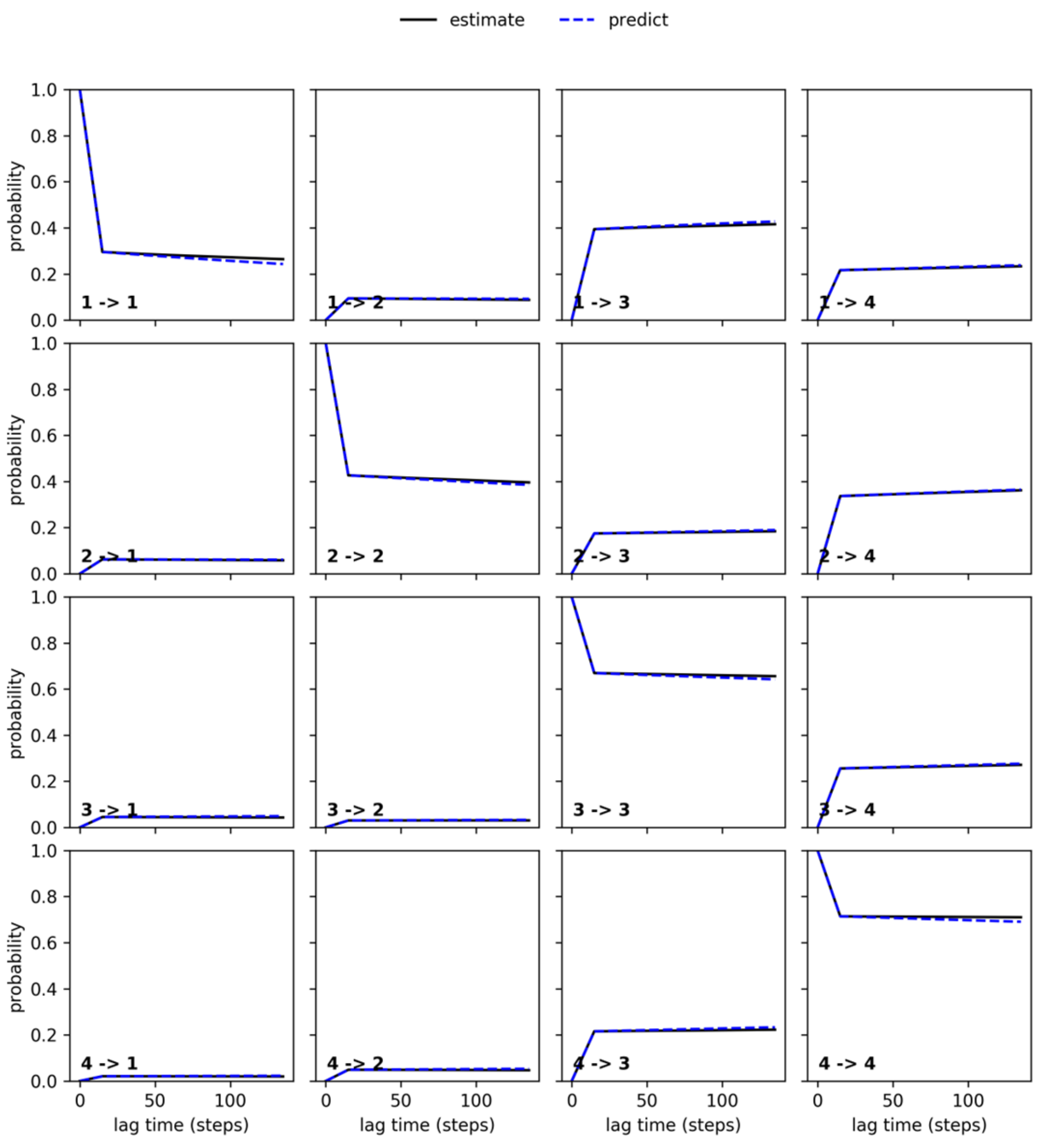

Figure S18. Chapman-Kolmogorov tests for replicate 3 of the *Cavity&Bulk* scheme. Plots show that MSMs correctly represent original MD simulation data.

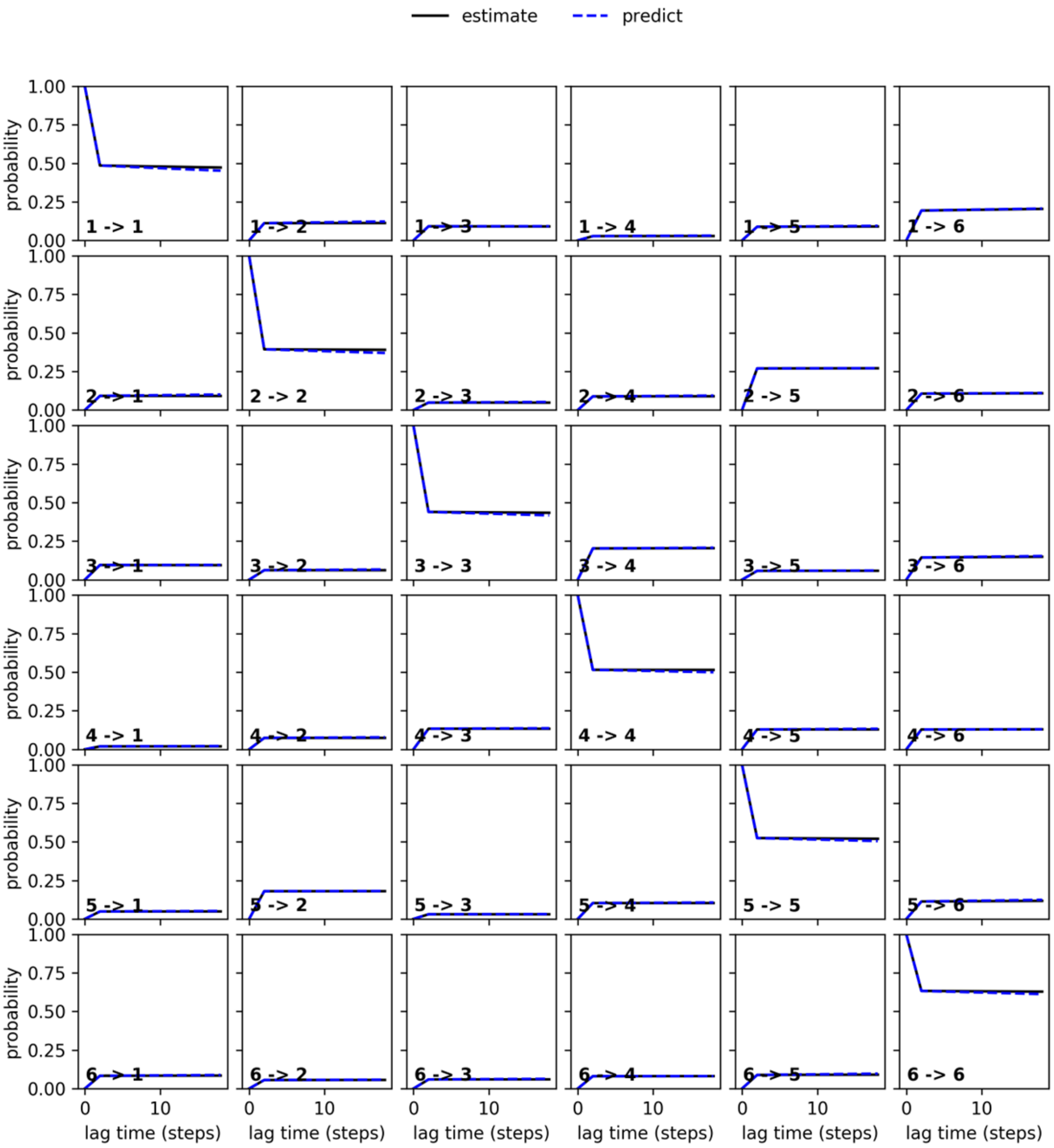

Figure S19. Chapman-Kolmogorov tests for replicate 1 of the *Tunnels* scheme. Plots show that MSMs correctly represent original MD simulation data.

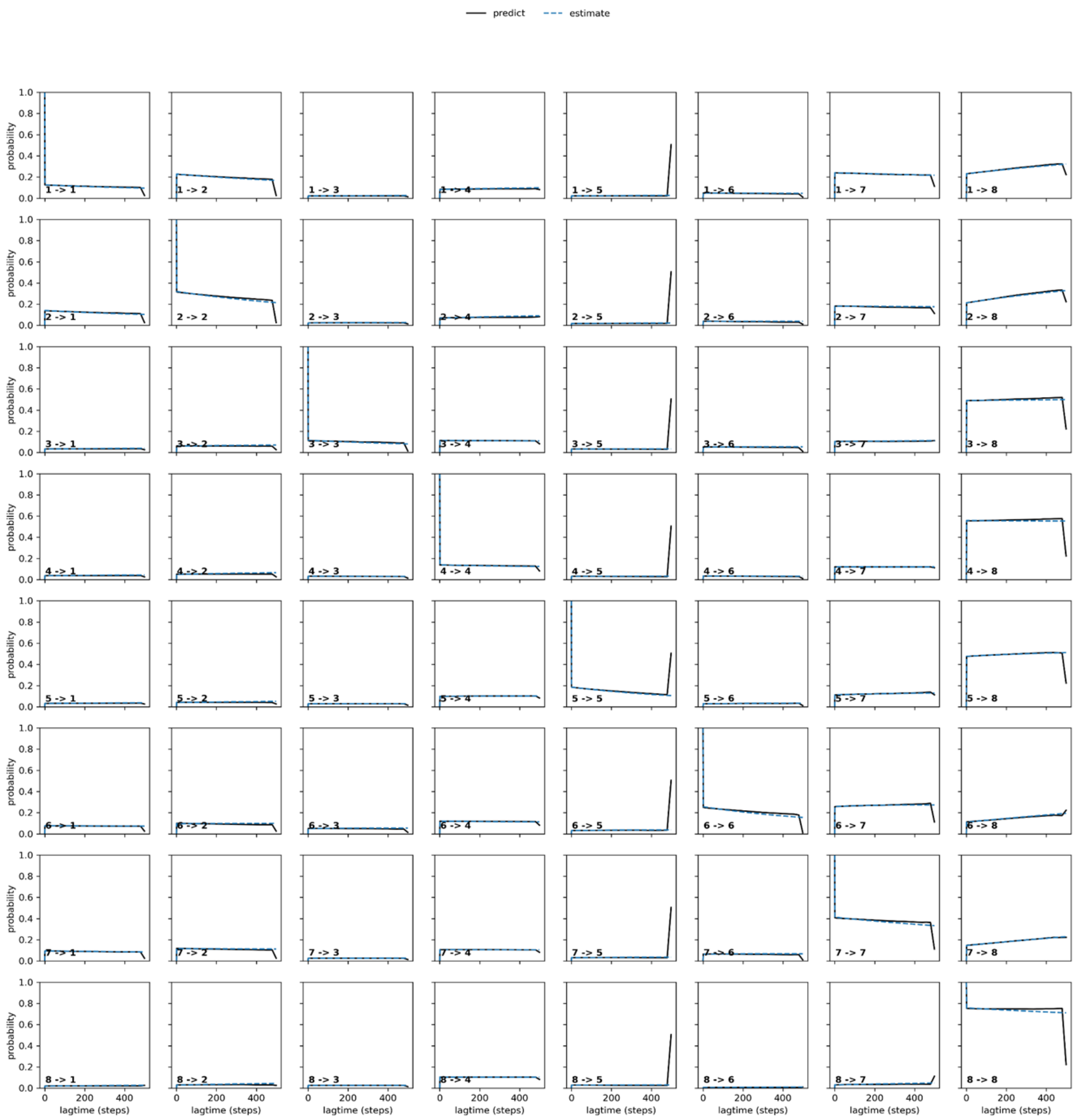

Figure S20. Chapman-Kolmogorov tests for replicate 2 of the *Tunnels* scheme. Plots show that MSMs correctly represent original MD simulation data.

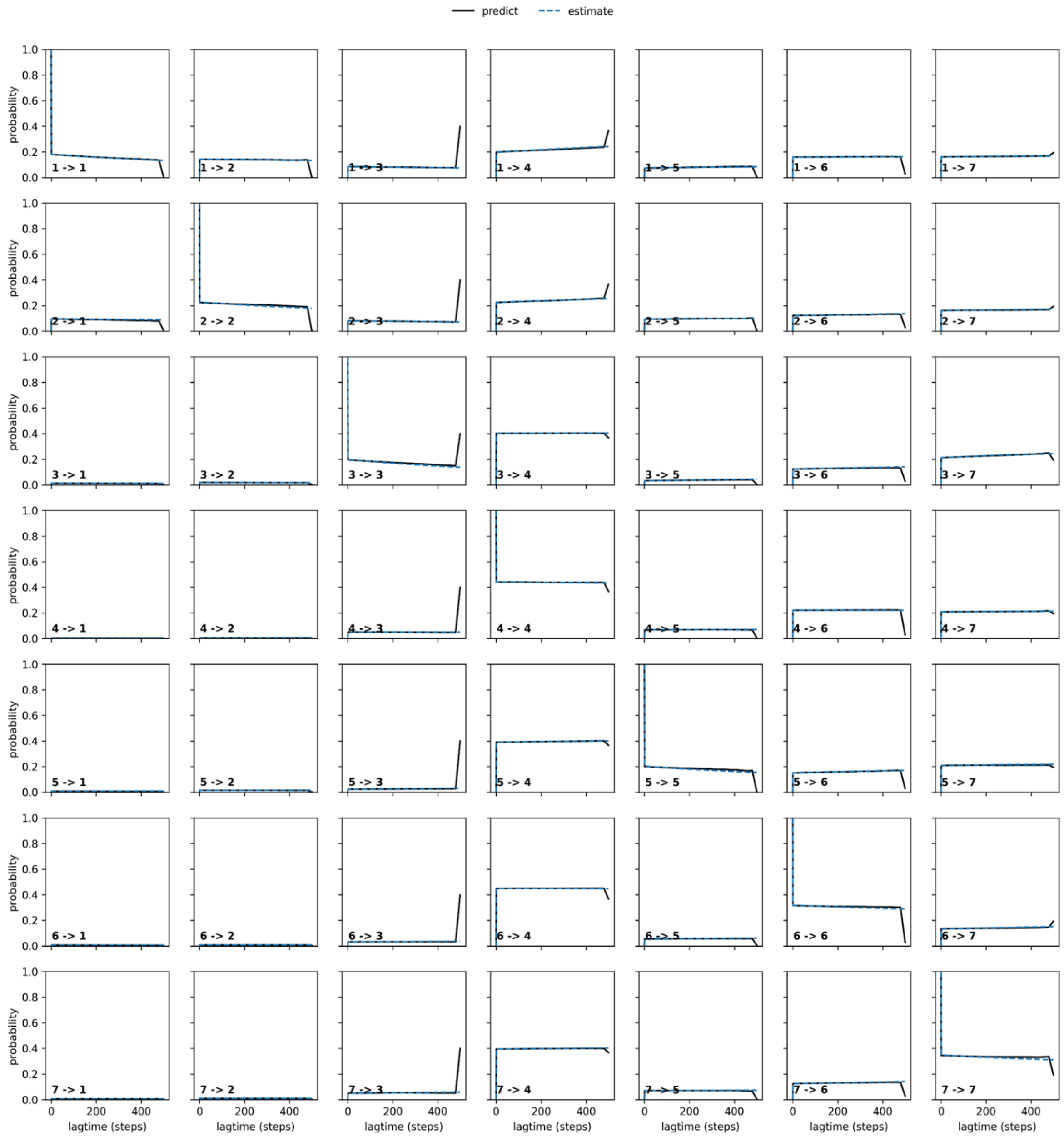

Figure S21. Chapman-Kolmogorov tests for replicate 3 of the *Tunnels* scheme. Plots show that MSMs correctly represent original MD simulation data.

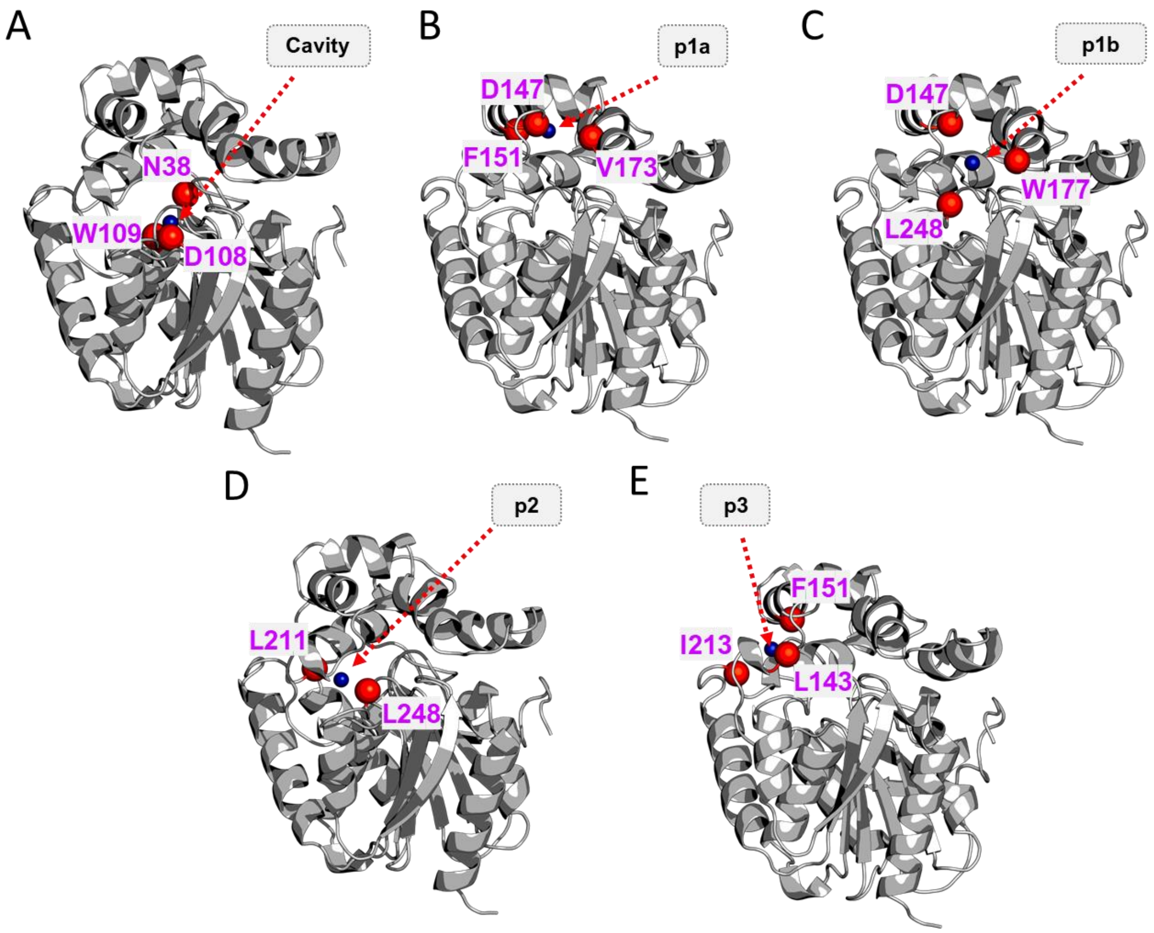

Figure S22. The COM of residues used to characterize DBE locations within the LinB86 structure. A) three catalytic residues defining the bottom of the active site cavity, B) residues defining the bottleneck of the *p1a* tunnel, C) residues defining the bottleneck of the *p1b* tunnel, D) residues defining the bottleneck of the *p2* tunnels, and E) residues defining the bottleneck of *p3* tunnel. The COMs are shown as blue spheres and the positions of the residues are defined by their CA atoms, shown as red spheres.

Table S2. Stability of the COM of residues used to characterize DBE localization within the LinB86 structure per scheme and replicate of ASMD simulations.

Schemes	Replicates	RMSF of COM [Å]				
		Cavity	*p1a* tunnel	*p1b* tunnel	*p2* tunnel	*p3* tunnel
Bulk	1	0.34	0.94	0.72	0.92	1.13
	2	0.36	0.59	0.64	0.73	0.64
	3	0.37	1.19	0.75	0.82	1.25
Cavity	1	0.35	1.27	0.83	0.93	1.36
	2	0.55	0.84	0.72	0.85	1.04
	3	0.32	1.27	0.69	0.55	1.19
Cavity&Bulk	1	0.32	0.99	0.84	1.09	1.19
	2	0.65	0.74	0.65	0.83	0.90
	3	0.53	0.93	0.72	1.04	1.21
Tunnels	1	1.12	1.01	0.75	1.10	1.21
	2	0.35	0.96	0.80	1.06	1.16
	3	0.36	0.83	0.76	1.18	0.96

Table S3. The number of ASMD simulations successfully completed per scheme and replicate.

Schemes	Replicates	Number of simulations	Total simulation time [μs]
Bulk	1	898	44.90
	2	900	45.00
	3	899	44.95
Cavity	1	888	44.40
	2	900	45.00
	3	897	44.85
Cavity&Bulk	1	899	44.95
	2	900	45.00
	3	891	44.55
Tunnels	1	886	44.30
	2	888	44.40
	3	897	44.85

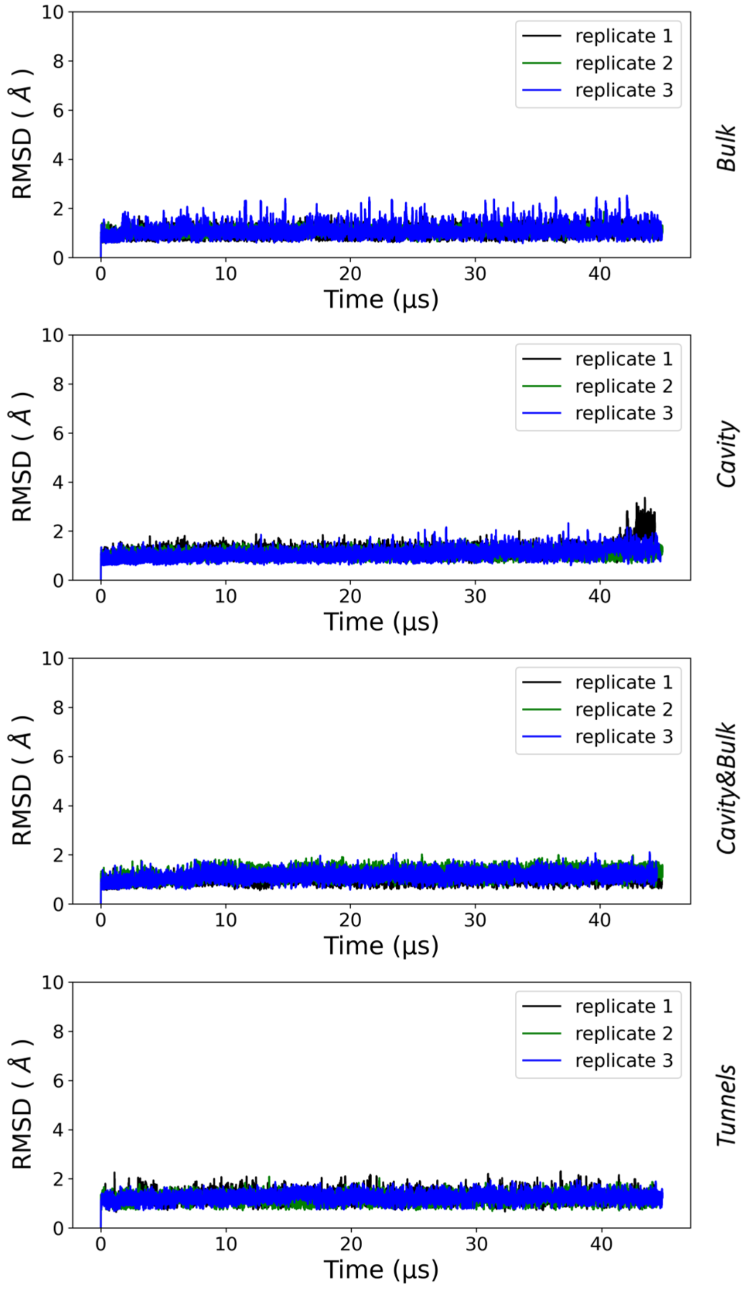

Figure S23 RMSD calculated from ASMD simulations. The individual trajectories in each replicate were joined into a single aligned trajectory and deviation in position of C_α atoms of entire protein except the first 10 residues forming the unstructured N-terminus (Figure S24) was analyzed.

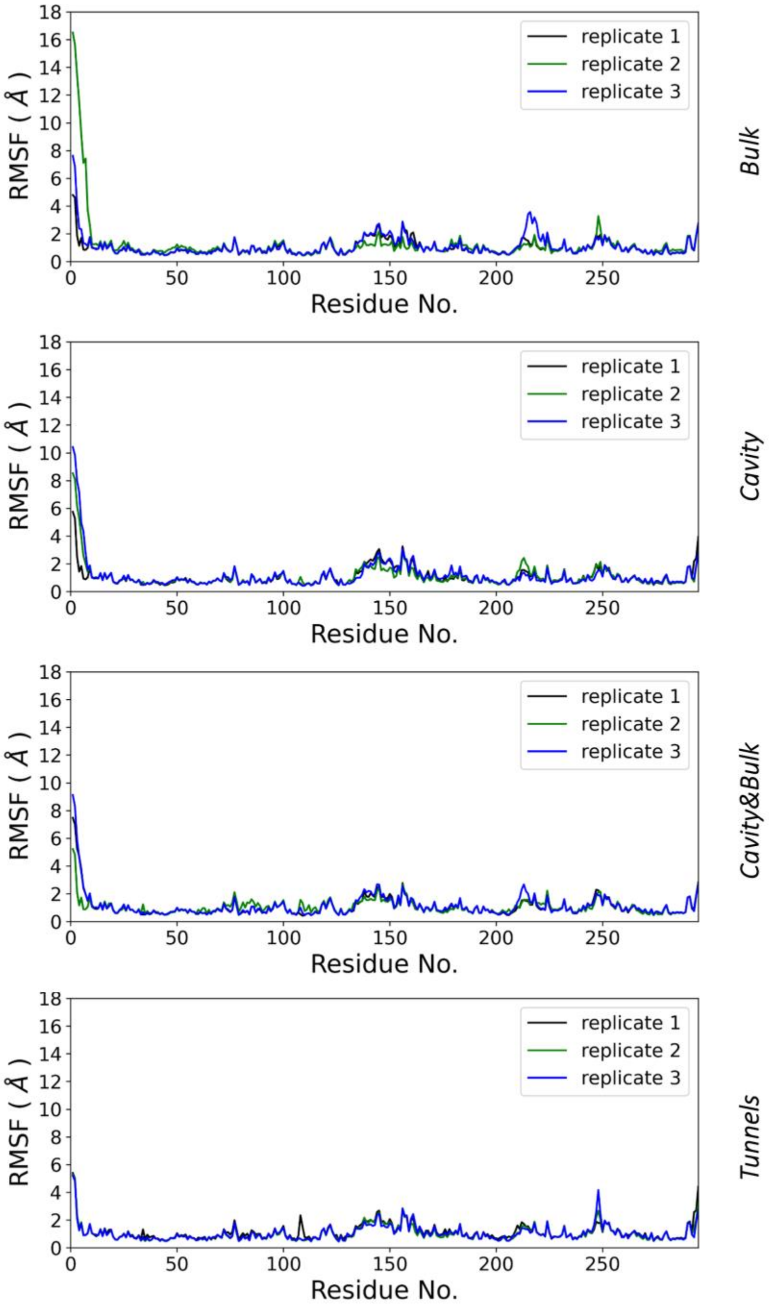

Figure S24 RMSF calculated from ASMD simulations. The individual trajectories in each replicate were joined into a single aligned trajectory and fluctuation of all atoms in each residue was analyzed.

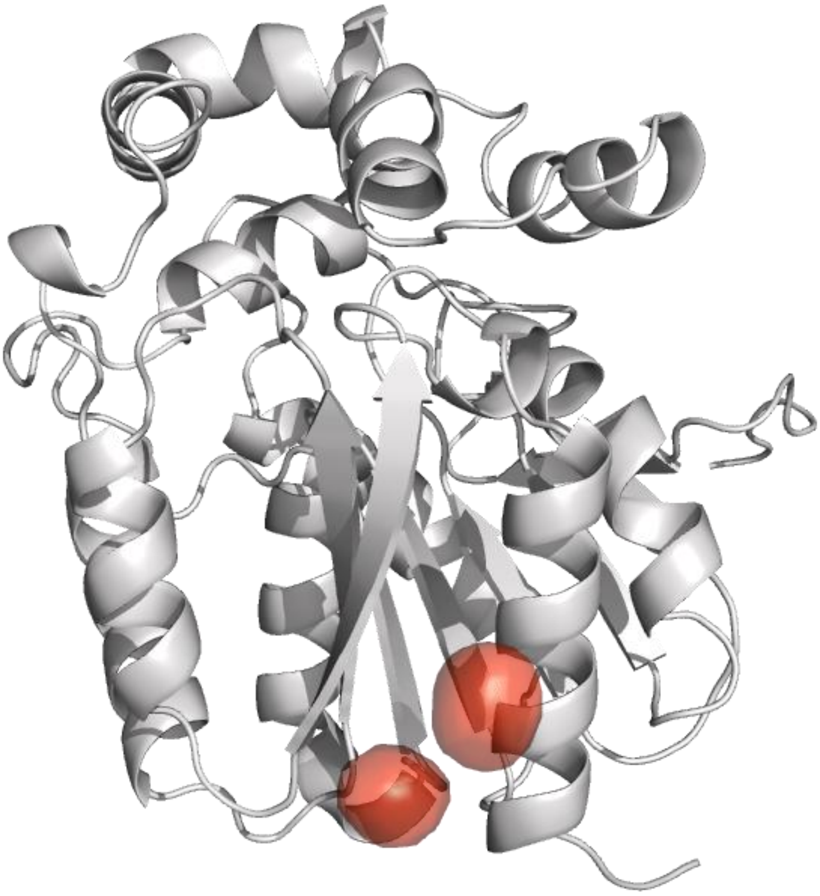

Figure S25. Region preferentially occupied by DBE molecule at approximately 25 Å-distance from the catalytic residues in *Bulk* scheme replicate2. Protein structure is shown as a gray cartoon while the region occupied by DBE molecule in 1 % structures with DBE molecule having distance to catalytic residues between 25 and 26 Å is shown as red surface. The distance is measured to the COM of catalytic residues (Figure S22A).

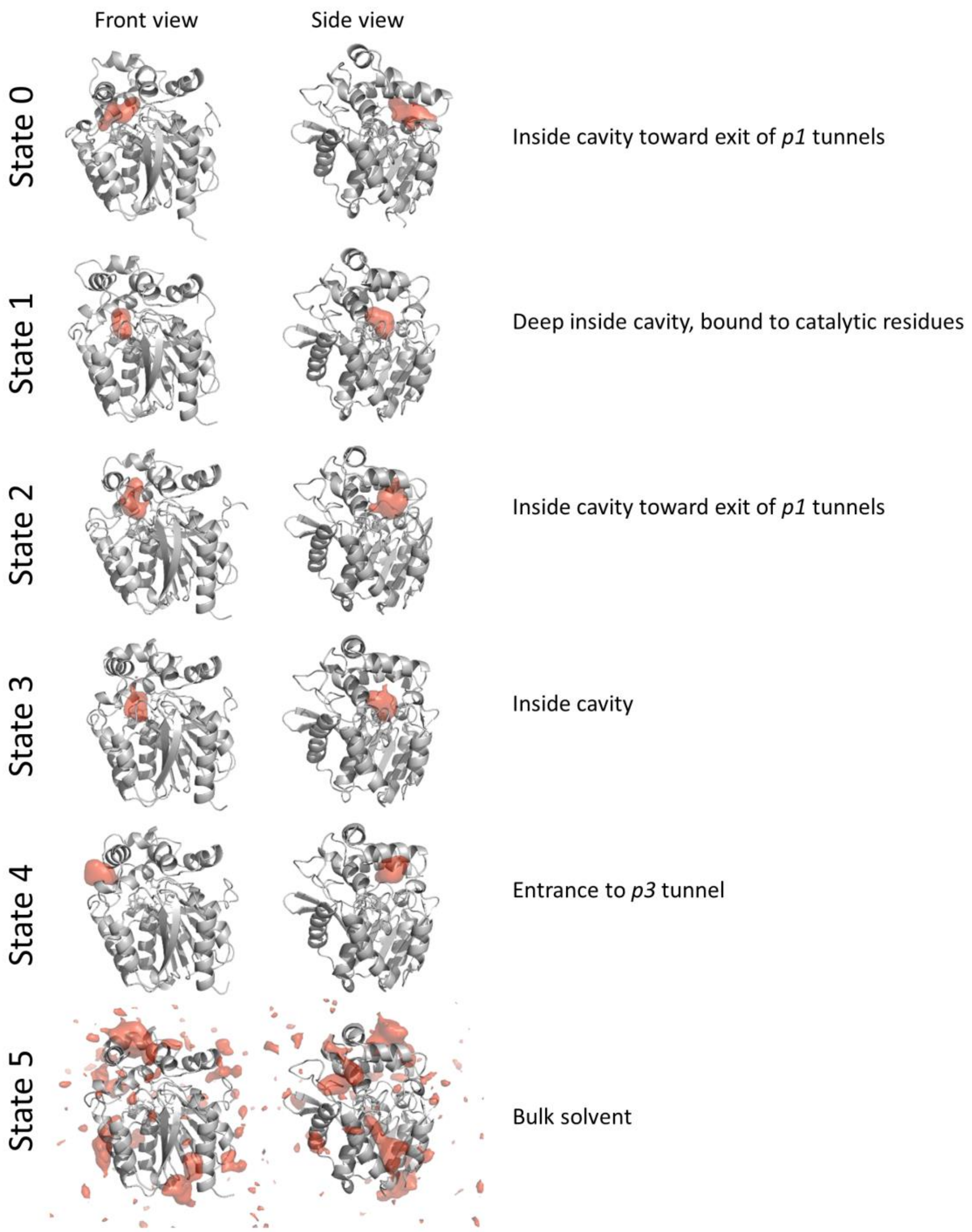

Figure S26. Overview metastable states derived from MSM of *Cavity* scheme replicate1. Protein structure is shown as a gray cartoon while the region occupied by DBE molecule in 20 % (1 % for bulk solvent state) of 1000 structures representing given metastable state is shown as red surface.

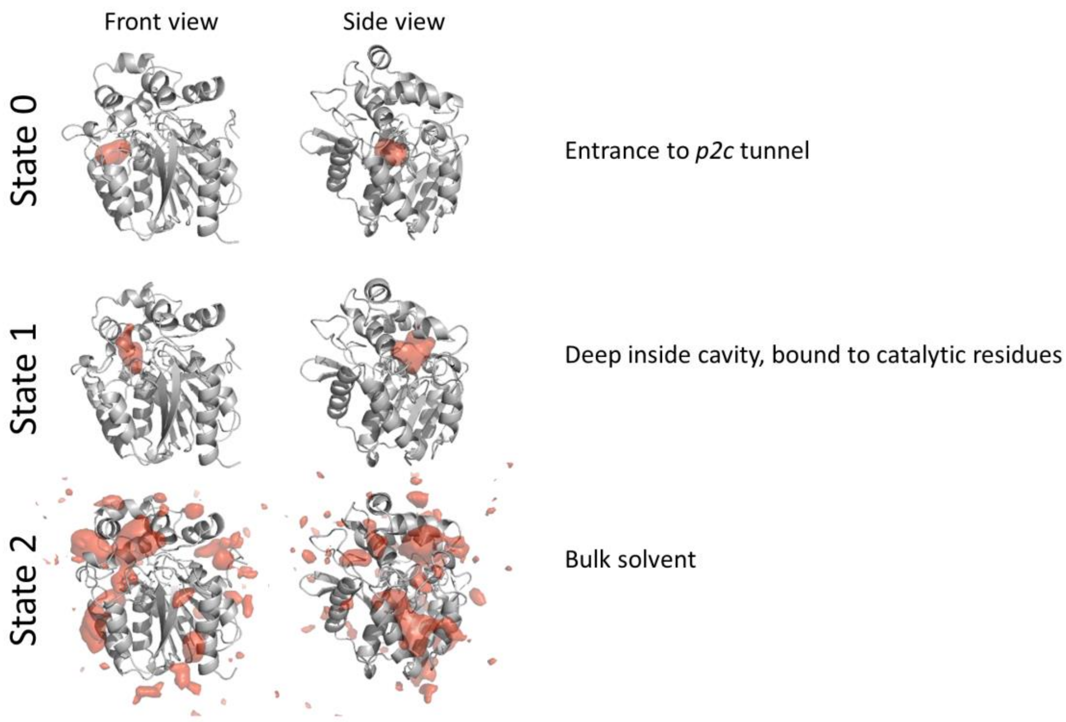

Figure S27. Overview metastable states derived from MSM of *Cavity* scheme replicate2. Protein structure is shown as a gray cartoon while the region occupied by DBE molecule in 20 % (1 % for bulk solvent state) of 1000 structures representing given metastable state is shown as red surface.

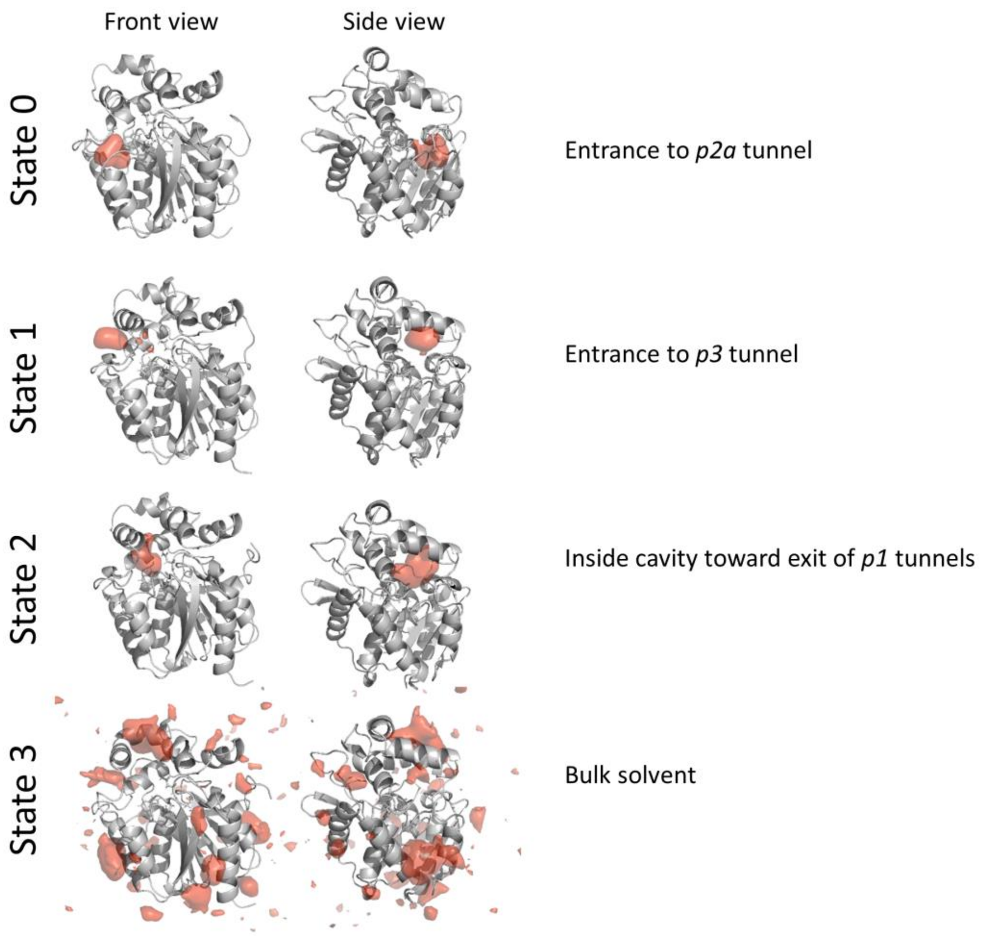

Figure S28. Overview metastable states derived from MSM of *Cavity* scheme replicate3. Protein structure is shown as a gray cartoon while the region occupied by DBE molecule in 20 % (1 % for bulk solvent state) of 1000 structures representing given metastable state is shown as red surface.

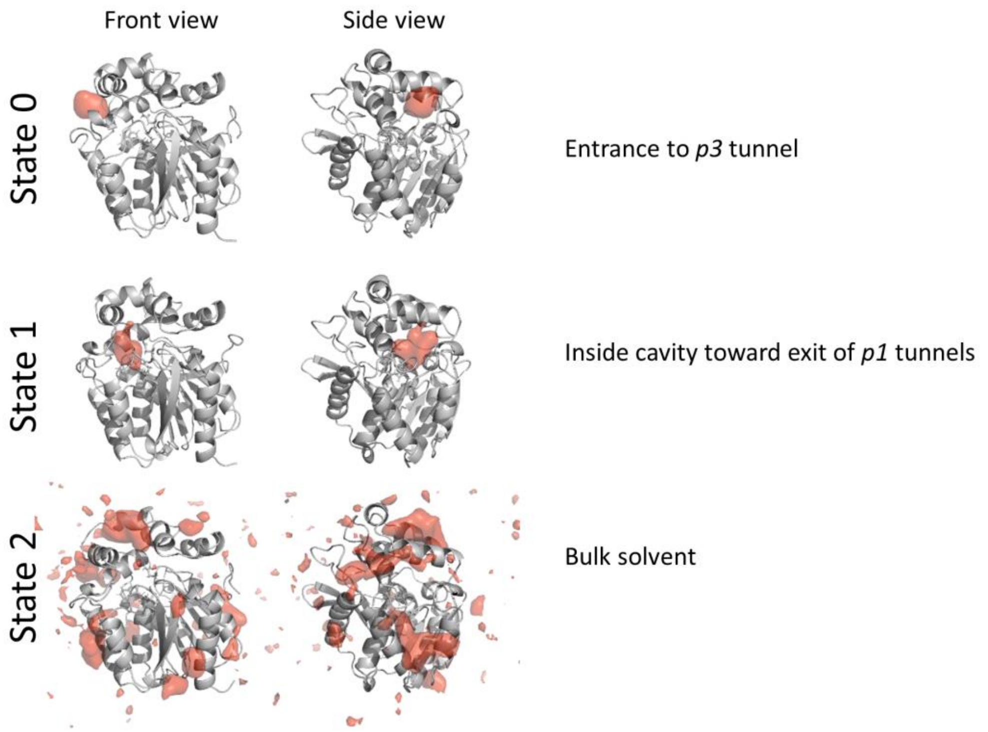

Figure S29. Overview metastable states derived from MSM of *Cavity&Bulk* scheme replicate1. Protein structure is shown as a gray cartoon while the region occupied by DBE molecule in 20 % (1 % for bulk solvent state) of 1000 structures representing given metastable state is shown as red surface.

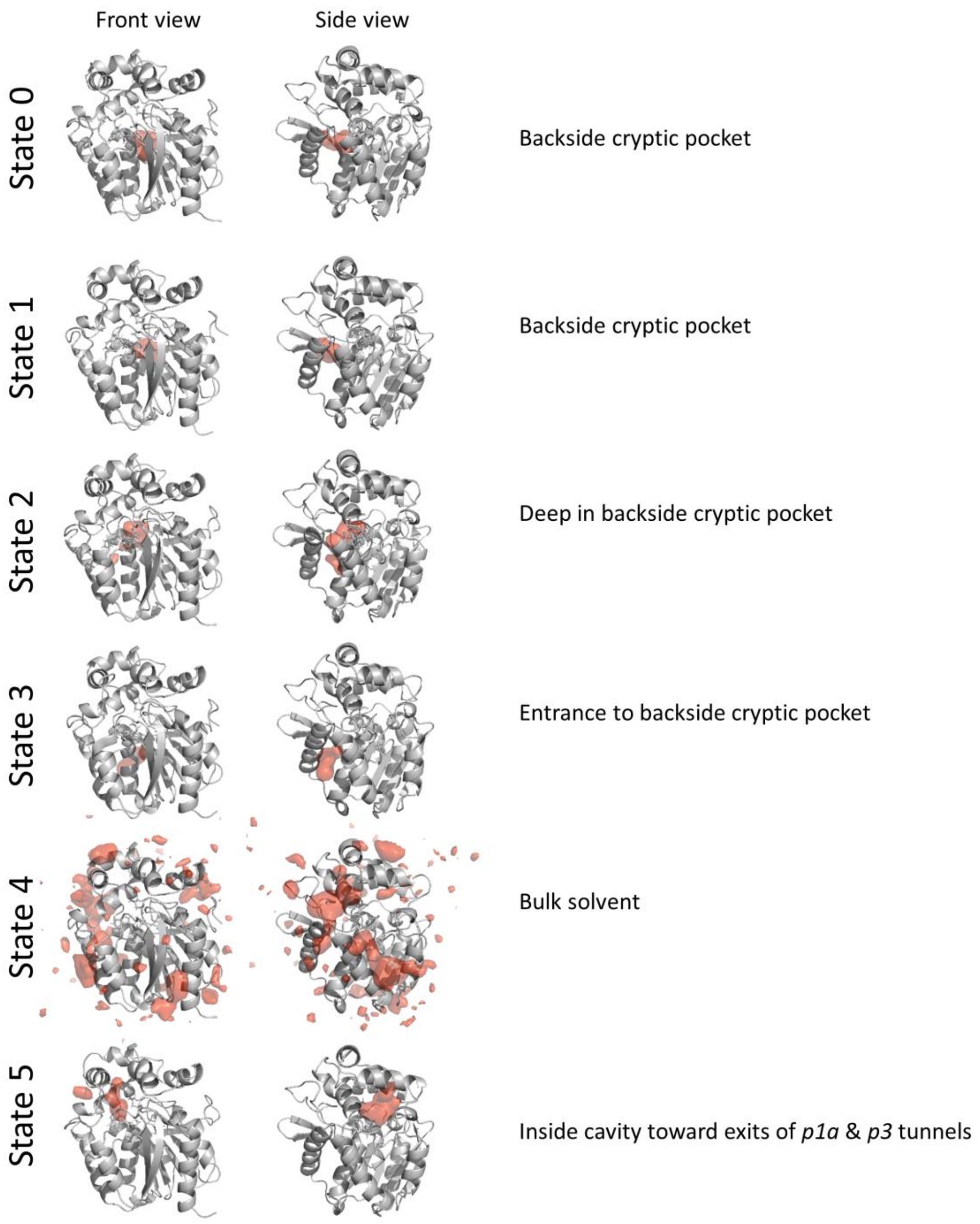

Figure S30. Overview metastable states derived from MSM of *Cavity&Bulk* scheme replicate2. Protein structure is shown as a gray cartoon while the region occupied by DBE molecule in 20 % (1 % for bulk solvent state) of 1000 structures representing given metastable state is shown as red surface.

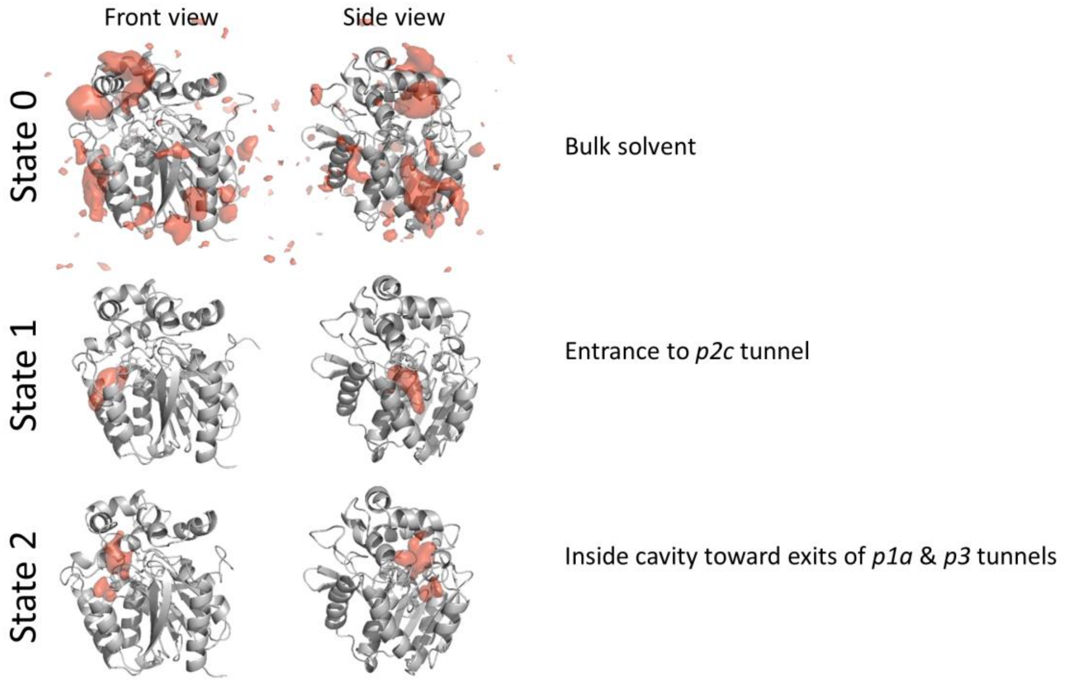

Figure S31. Overview metastable states derived from MSM of *Cavity&Bulk* scheme replicate3. Protein structure is shown as a gray cartoon while the region occupied by DBE molecule in 20 % (1 % for bulk solvent state) of 1000 structures representing given metastable state is shown as red surface.

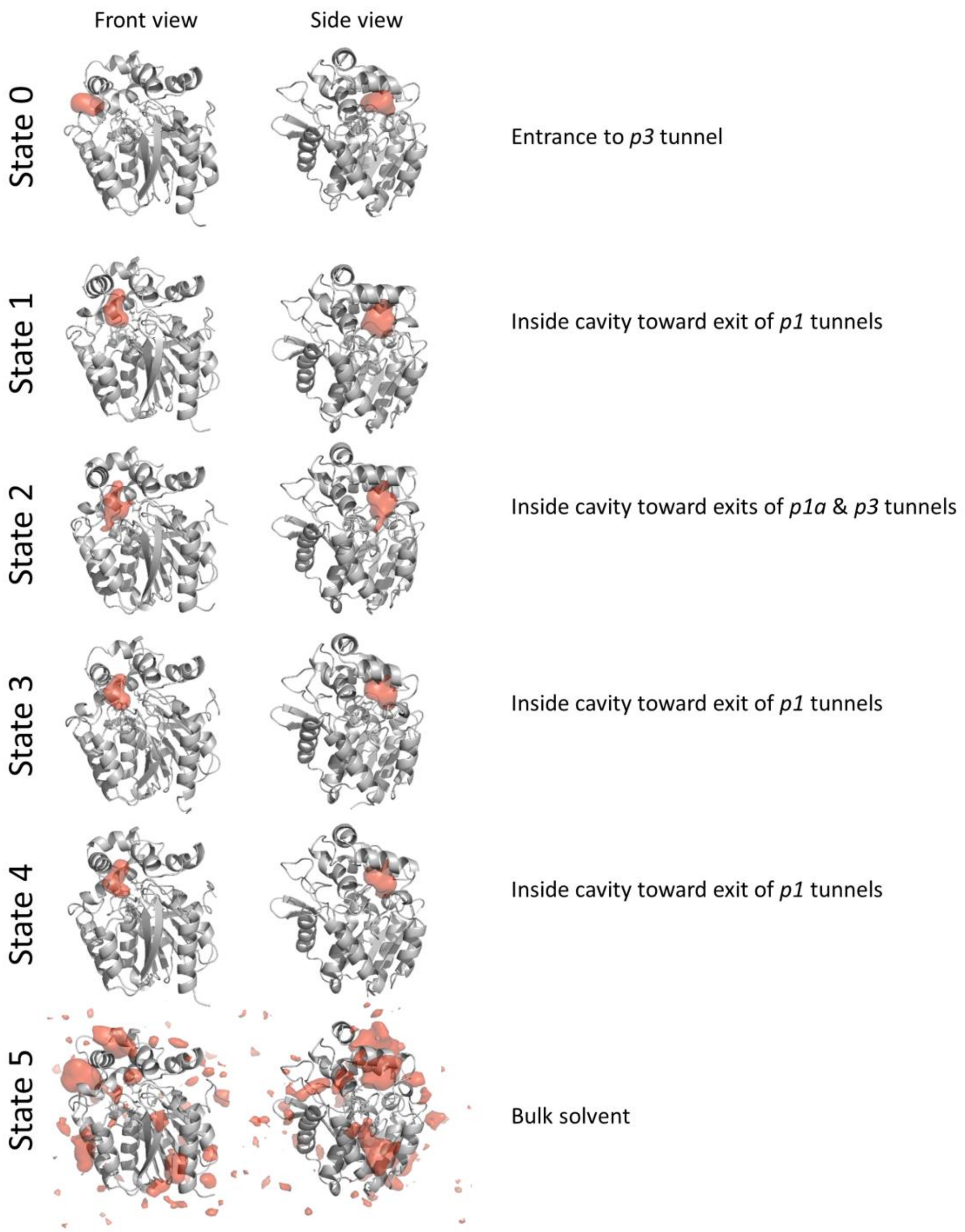

Figure S32. Overview metastable states derived from MSM of *Tunnels* scheme replicate1. Protein structure is shown as a gray cartoon while the region occupied by DBE molecule in 20 % (1 % for bulk solvent state) of 1000 structures representing given metastable state is shown as red surface.

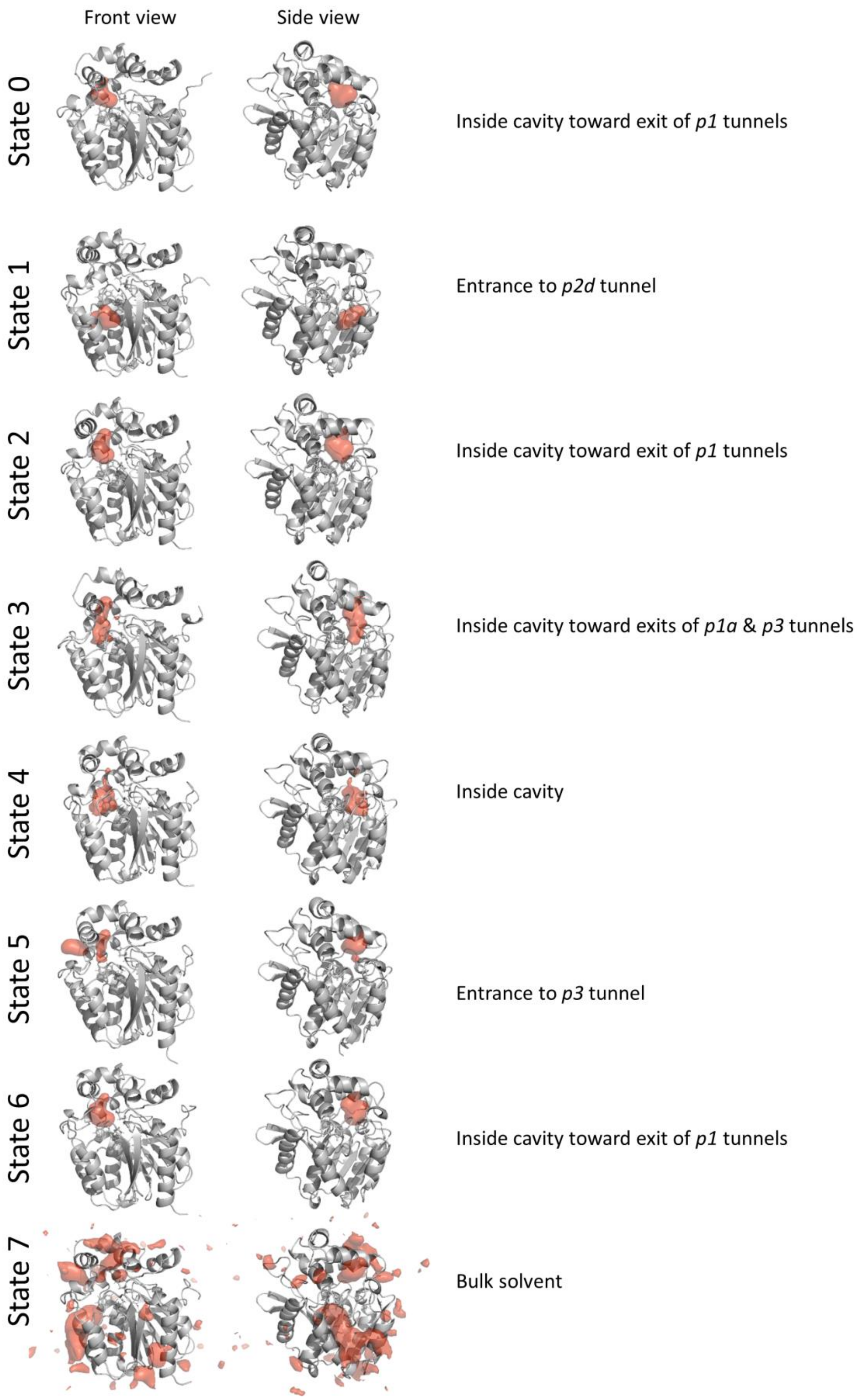

Figure S33. Overview metastable states derived from MSM of *Tunnels* scheme replicate2. Protein structure is shown as a gray cartoon while the region occupied by DBE molecule in 20 % (1 % for bulk solvent state) of 1000 structures representing given metastable state is shown as red surface.

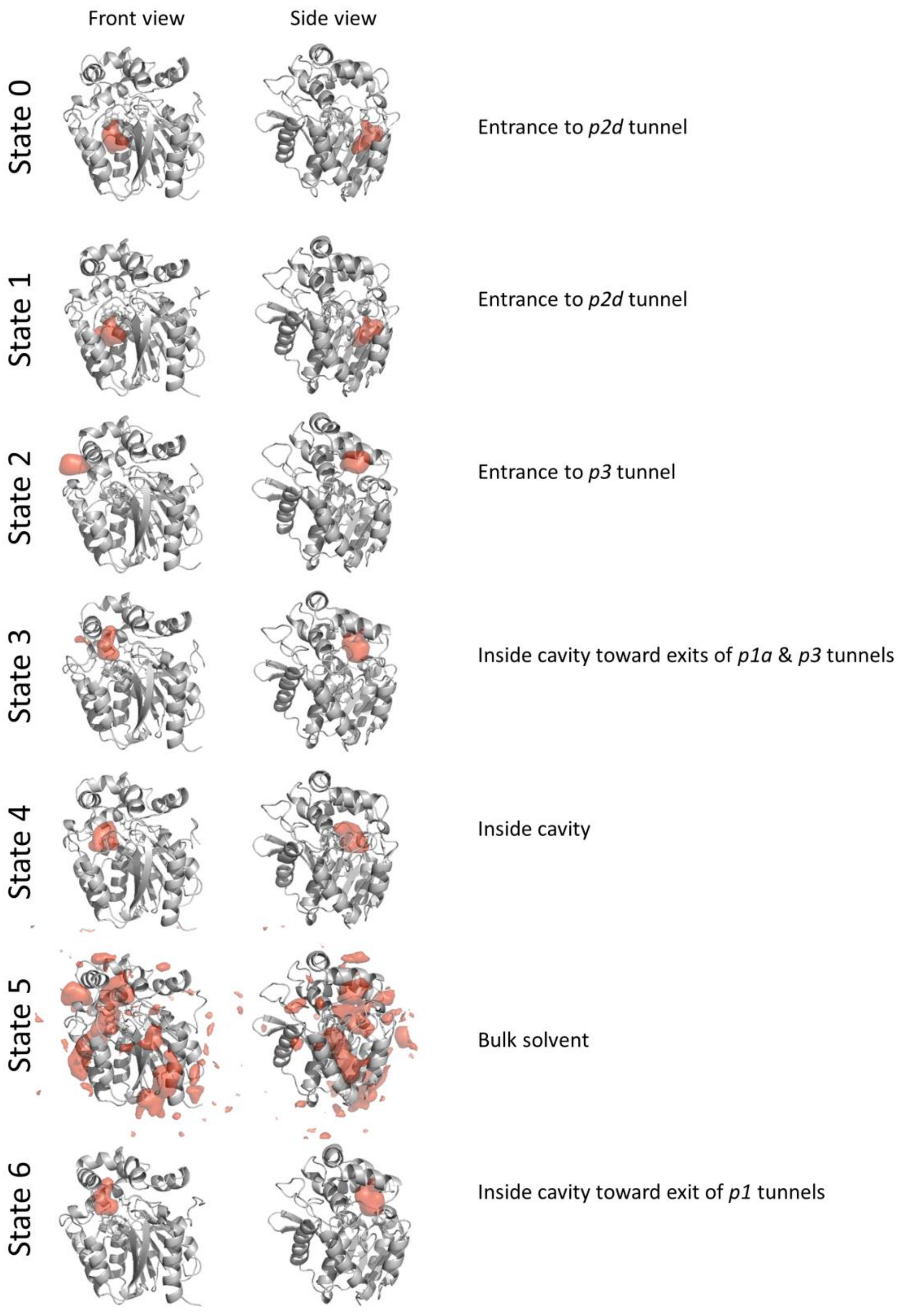

Figure S34. Overview metastable states derived from MSM of *Tunnels* scheme replicate3. Protein structure is shown as a gray cartoon while the region occupied by DBE molecule in 20 % (1 % for bulk solvent state) of 1000 structures representing given metastable state is shown as red surface.

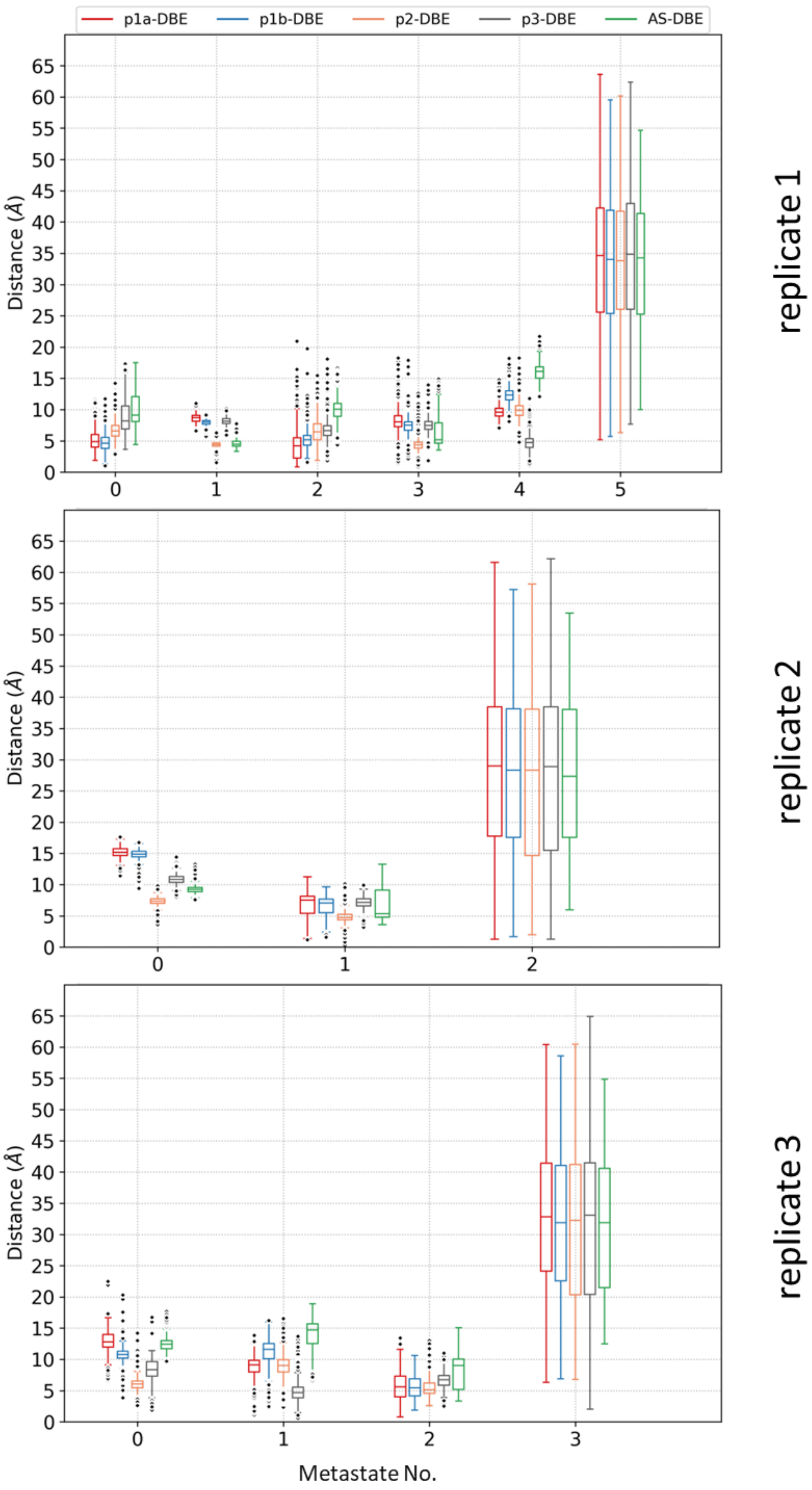

Figure S35. Characteristic distances of representative structures of metastable states from the *Cavity* scheme.

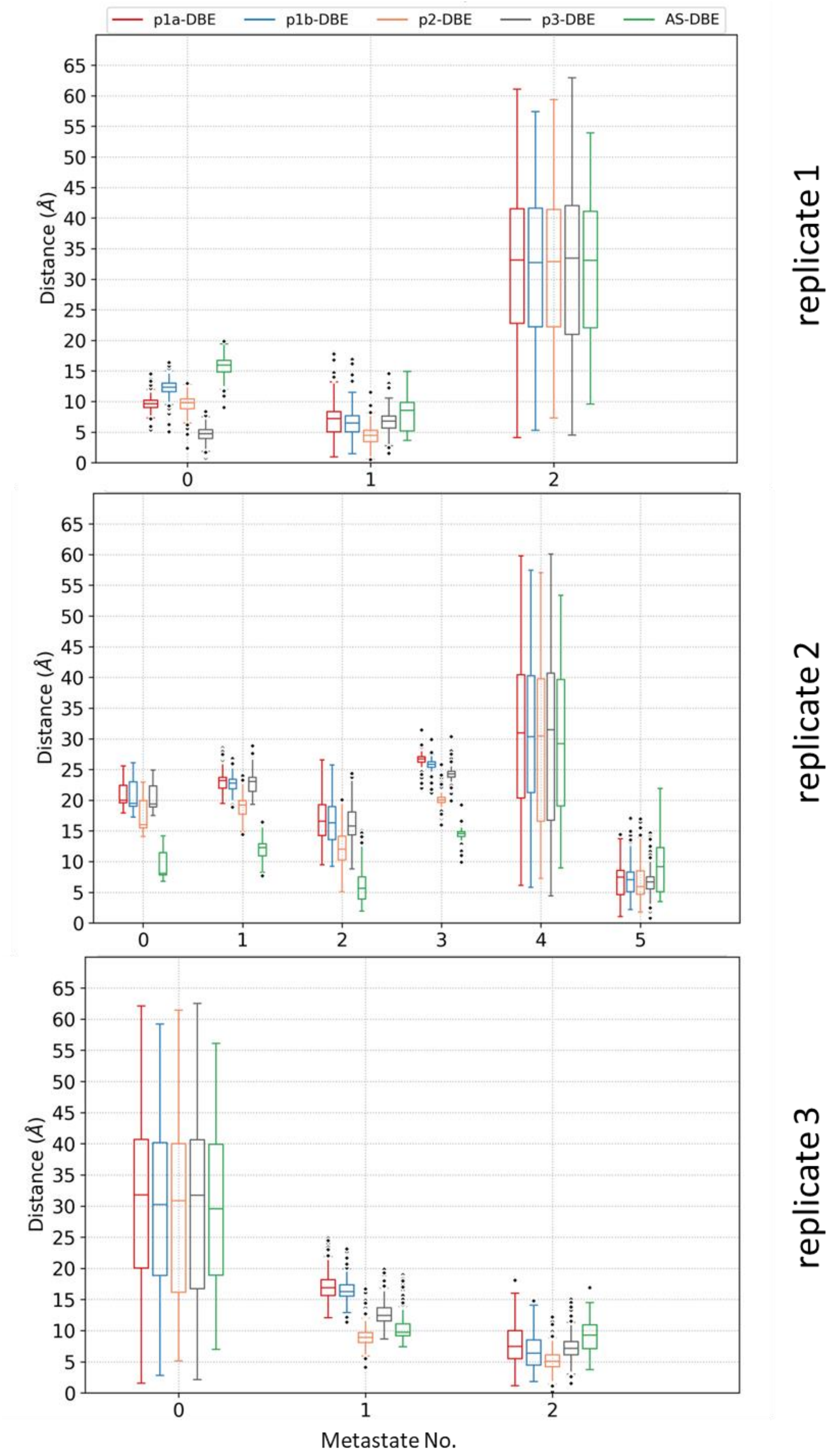

Figure S36. Characteristic distances of representative structures of metastable states from the *Cavity&Bulk* scheme.

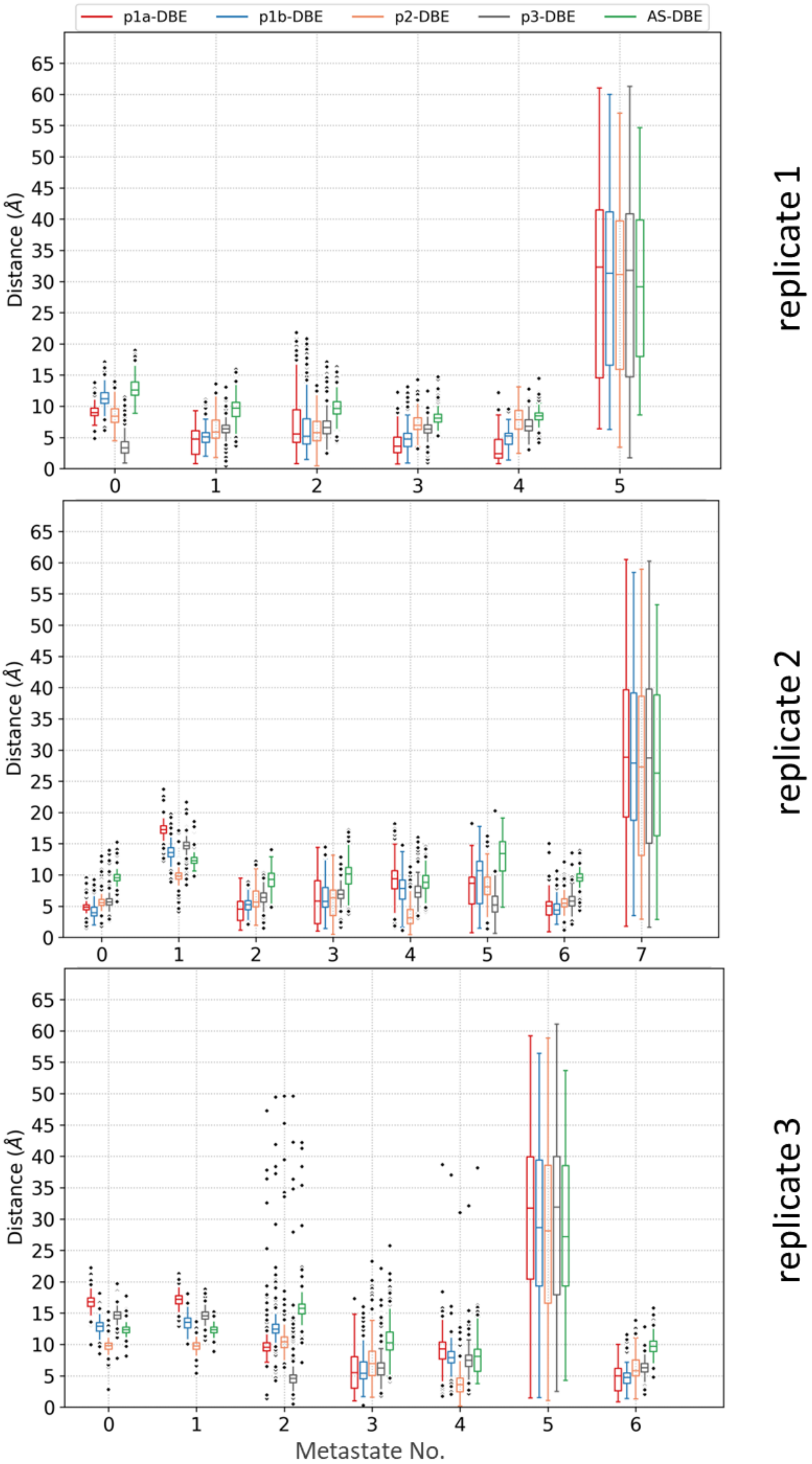

Figure S37. Characteristic distances of representative structure of metastable states from the *Tunnels* scheme.

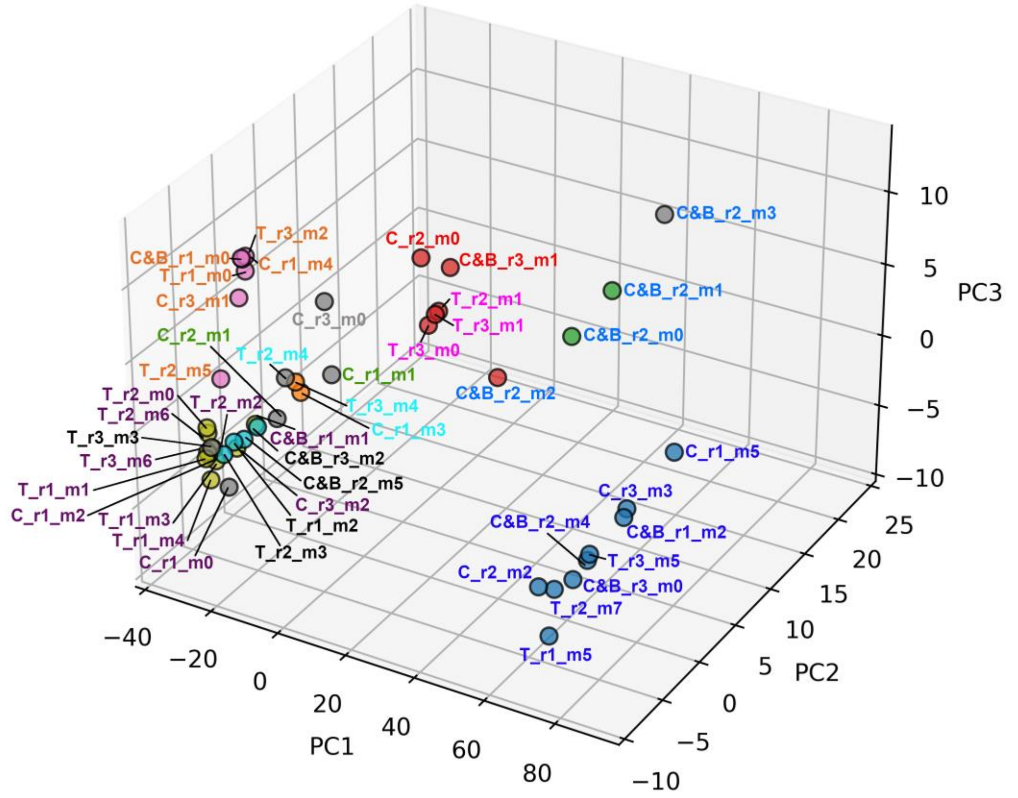

ULS1	ULS2	ULS3	ULS4	ULS5	ULS6	ULS7	ULS8	ULS9	ULS10
C_r1_m5	C_r1_m1	T_r2_m4	C_r1_m0	C&B_r3_m2	T_r3_m2	C_r3_m0	T_r2_m1	C&B_r3_m1	C&B_r2_m3
C_r3_m3	C_r2_m1	T_r3_m4	T_r1_m4	C&B_r2_m5	C_r1_m4		T_r3_m1	C_r2_m0	C&B_r2_m1
C&B_r1_m2		C_r1_m3	T_r1_m3	T_r1_m2	C&B_r1_m0		T_r3_m0		C&B_r2_m0
C&B_r2_m4			T_r2_m0	T_r2_m3	T_r1_m0				C&B_r2_m2
T_r3_m5			T_r2_m6	T_r3_m3	C_r3_m1				
C&B_r3_m0			T_r2_m2		T_r2_m5				
T_r2_m7			C&B_r1_m1						
C_r2_m2			C_r3_m2						
T_r1_m5			T_r3_m6						
			T_r1_m1						
			C_r1_m2						

Figure S38. Clustering of metastable states from all MSM analyses into unified ligand states (ULS). The location of individual metastable states (Figures S26-S34) according to their fingerprints of characteristic distances (Figures S35-S37) in space formed by three principal components (PC) from the PC analysis. The disks are colored based on the cluster membership from the HDBSCAN method, with the gray disk corresponding to cluster composed of outliers. The metastable states are labeled according to the seeding scheme (C-*Cavity*, C&B-*Cavity&Bulk*, and T-*Tunnels*) followed by replicate (r1-3) and the metastable state number (m0-7) and colored by their assignment to the respective ULS.

Table S4. Configurations in metastable states with DBE within 5 Å from the catalytic residues of LinB86.

Scheme	Replicate	Percent of configurations Metastable state No.							
		**0**	**1**	**2**	**3**	**4**	**5**	**6**	**7**
Cavity	1	1	**78**	4	42	0	**0**	-	-
	2	0	**36**	**0**	-	-	-	-	-
	3	0	0	**17**	**0**	-	-	-	-
Cavity&Bulk	1	0	**22**	**0**	-	-	-	-	-
	2	0	0	0	0	**0**	**24**	-	-
	3	**0**	0	**10**	-	-	-	-	-
Tunnels	1	0	1	**3**	0	0	**0**	-	-
	2	0	0	0	2	**6**	0	0	**0**
	3	0	0	0	1	**14**	**0**	0	-

Metastates used as bound and unbound states are bolded.

Table S5. Dissociation and association rates of DBE from/to LinB86 active site derived from MSMs.

Parameter	Scheme	Replicate			Average	S.E.M	Relative error [%]
		**1**	**2**	**3**			
k_{on} $[M^{-1}\cdot s^{-1}]\cdot 10^8$	*Cavity*	3.55	2.45	3.30	3.1	0.3	11
	Cavity&Bulk	2.23	2.71	4.02	3.0	0.5	18
	Tunnels	1.26	3.20	4.62	3.0	1.0	32
k_{off} $[s^{-1}]\cdot 10^6$	*Cavity*	4.54	3.15	1.22	3.0	1.0	32
	Cavity&Bulk	3.30	4.92	3.72	4.0	0.5	12
	Tunnels	0.72	3.23	2.75	2.2	0.8	34
k_{off}/k_{on} $[M]\cdot 10^{-3}$	*Cavity*	12.80	12.80	3.70	9.8	3.0	31
	Cavity&Bulk	14.80	18.20	9.27	14.1	2.6	18
	Tunnels	5.69	10.10	5.95	7.2	1.4	20

S.E.M – standard error of mean

Table S6. Sensitivity of calculated k_{off}/k_{on} values to the bound state selection.

Scheme/replicate	Bound state		k_{off}/k_{on} [M] · 10^{-3}
	**Metastate No.**	**Unified ligand state**	
Cavity/replicate1	**1**	**2**	**12.8**
	3	3	9.8
	2	4	7.6
Tunnels/replicate1	1	4	9.0
	**2**	**5**	**5.7**
Tunnels/replicate2	**4**	**3**	**10.1**
	3	5	7.4
Tunnels/replicate3	**4**	**3**	**6.0**
	3	5	7.5

Bound states with largest percent of configurations with DBE molecule within 5 Å from the catalytic residues of LinB86 (Table S4) are bolded.

Table S7. T-test statistics for difference of means between experimental and calculated k_d values.

Scheme	Calculated			Experimental			p-value	95% confidence interval of the difference
	Average	S.E.M	N	Average	S.E.M	N		
Cavity	10	3	3				0.0190	1.49 - 12.51
Cavity&Bulk	14	3	3	17	1	7	0.2449	-2.51 - 8.51
Tunnels	7	1	3				0.0004	6.10 - 13.90

S.E.M – standard error of mean

Table S8. Number of substrate migration events obtained from TransportTools analyses per schemes and replicates.

Schemes	Replicates	Total events	Assigned to tunnel				Unassigned events
			p1a	***p1b***	***p2***	***p3***	
Cavity	1	11	0	0	0	0	11
	2	40	11	14	1	7	7
	3	7	0	0	0	0	7
Cavity&Bulk	1	5	0	0	0	0	5
	2	1	0	0	0	0	1
	3	3	0	0	0	1	2
Tunnels	1	6	0	0	0	1	5
	2	6	0	1	0	0	5
	3	10	0	0	0	0	10

Table S9. Per scheme and tunnel counts and detailed statistics of bottlenecks' crossing by DBE molecule.

Tunnel	rep1	rep2	rep3	sum/tunnel	avg/tunnel	std/tunnel
Scheme Bulk						
p1a	358	17	156	531	177.0	171.5
p1b	527	4	293	824	274.7	262.0
p2	614	15	404	1033	344.3	303.9
p3	216	8	81	305	101.7	105.5
unknown	13	4	11	28	9.3	4.7
mixed	92	1	61	154	51.3	46.3
sum/rep	1820	49	1006			
sum/scheme	2875					
avg/scheme	958.3					
std/scheme	886.5					
Scheme Cavity						
p1a	626	231	418	1275	425.0	197.6
p1b	525	160	883	1568	522.7	361.5
p2	739	251	1084	2074	691.3	418.5
p3	261	62	320	643	214.3	135.2
unknown	40	10	39	89	29.7	17.0
mixed	95	20	165	280	93.3	72.5
sum/rep	2286	734	2909			
sum/scheme	5929					
avg/scheme	1976.3					
std/scheme	1120.1					
Scheme Cavity&Bulk						
p1a	241	113	131	485	161.7	69.3
p1b	239	80	689	1008	336.0	315.9
p2	700	115	970	1785	595.0	437.1
p3	143	71	155	369	123.0	45.4
unknown	6	39	25	70	23.3	16.6
mixed	47	16	84	147	49.0	34.0
sum/rep	1376	434	2054			
sum/scheme	3864					
avg/scheme	1288.0					
std/scheme	813.6					
Scheme Tunnels						
p1a	363	306	261	930	310.0	51.1
p1b	931	425	854	2210	736.7	272.6
p2	1071	726	1391	3188	1062.7	332.6
p3	280	134	199	613	204.3	73.1
unknown	35	12	52	99	33.0	20.1
mixed	129	77	211	417	139.0	67.6
sum/rep	2809	1680	2968			
sum/scheme	7457					
avg/scheme	2485.7					
std/scheme	702.2					

References

(1) Case, D. A.; Walker, R. C.; Cheatham, T. E.; Simmerling, C.; Roitberg, A.; Merz, K. M.; Luo, R.; Darden, T. Amber 18. *University of California, San Francisco.* **2018**.

(2) Zwanzig, R. Nonlinear Generalized Langevin Equations. *J. Stat. Phys.* **1973**, *9*, 215-220.

(3) Hopkins, C. W.; Le Grand, S.; Walker, R. C.; Roitberg, A. E. Long-Time-Step Molecular Dynamics through Hydrogen Mass Repartitioning. *J. Chem. Theory. Comput.* **2015**, *11*, 1864-1874.

(4) Chovancova, E.; Pavelka, A.; Benes, P.; Strnad, O.; Brezovsky, J.; Kozlikova, B.; Gora, A.; Sustr, V.; Klvana, M.; Medek, P.; Biedermannova, L.; Sochor, J.; Damborsky, J. CAVER 3.0: A Tool for the Analysis of Transport Pathways in Dynamic Protein Structures. *PLoS Comput. Biol.* **2012**, *8*, 23-30.

(5) Pavelka, A.; Sebestova, E.; Kozlikova, B.; Brezovsky, J.; Sochor, J.; Damborsky, J. CAVER: Algorithms for Analyzing Dynamics of Tunnels in Macromolecules. *IEEE/ACM Trans. Comput. Biol. Bioinform.* **2016**, *13*, 505-517.

(6) Vavra, O.; Filipovic, J.; Plhak, J.; Bednar, D.; Marques, S. M.; Brezovsky, J.; Stourac, J.; Matyska, L.; Damborsky, J. CaverDock: A Molecular Docking-Based Tool to Analyse Ligand Transport through Protein Tunnels and Channels. *Bioinformatics* **2019**, *35*, 4986-4993.
